# *BMC Ecology* Image Competition 2015: the winning images

**DOI:** 10.1186/s12898-015-0053-9

**Published:** 2015-07-29

**Authors:** Catherine J Potenski, Ana Luz Porzecanski, Michel Baguette, Jean Clobert, David Hughes, Josef Settele

**Affiliations:** 1 BioMed Central, 233 Spring Street, New York, NY 10013 USA; 2 Center for Biodiversity and Conservation, American Museum of Natural History, Central Park West at 79th Street, New York, NY 10024 USA; 3 Institut de Systématique, Evolution et Biodiversité, Muséum National d’Histoire Naturelle (MNHN), UMR 7205, 75005 Paris, France; 4 Station d’Ecologie Experimentale du CNRS, 09200 Moulis, France; 5 Department of Entomology and Department of Biology, Center for Infectious Disease Dynamics, Pennsylvania State University, University Park, State College, PA USA; 6 Department of Community Ecology, Helmholtz Centre for Environmental Research-UFZ, Theodor-Lieser-Str. 4, 06120 Halle, Germany; 7 iDiv, German Centre for Integrative Biodiversity Research, Halle-Jena-Leipzig, Deutscher Platz 5e, 04103 Leipzig, Germany

## Abstract

For the third time, *BMC Ecology* is delighted to announce the winners of our Image Competition. This year featured entries from all over the world and showcased not only the creativity and talent of the participants, but also the exquisite beauty and diversity of our planet. We are pleased to present the winning selections of the editorial board of the journal and guest judge Dr. Ana Luz Porzecanski, as well as some highly commended images that are sure to impress.

**Electronic supplementary material:**

The online version of this article (doi:10.1186/s12898-015-0053-9) contains supplementary material, which is available to authorized users.

## Editorial

“Ecology” derives from the Greek word for “house”. Across the four oceans and seven continents, ecologists study the wondrous organisms, interactions and locations that, combined, make up our own house, the planet Earth. Whether cataloguing biodiversity, describing a new species interaction or modeling migratory patterns, ecologists contribute to our understanding of the world and our place in it. They are the connoisseurs of the beauty and mystery of our natural surroundings, so who better to act as field guides to show us our collective home?

*BMC Ecology* started an Image Competition for this very reason: to give ecologists the opportunity to share their perspective with the rest of us. Ecologists can then educate as they draw attention to some of the outstanding science being done, while featuring their research efforts in a visual, and fun, way. How does an ecologist view the world? You’ve come to the right place to find out.

Our past competitions have captured some of the aspects of this ecological outlook [1, 2], and we were eager to continue with that in 2015. We were absolutely thrilled to have Dr. Ana Luz Porzecanski on board to act as our guest judge for this year. Dr. Porzecanski is the Director of the Center for Biodiversity and Conservation at the American Museum of Natural History in New York, NY, USA [3]. Her work advances conservation awareness, biodiversity research and community outreach and she is a passionate advocate for ecological science. Her expertise and experience as a scientist and educator make her an excellent judge of the submissions to the image competition. The editorial board of *BMC Ecology* kindly lent their support and enthusiasm for ecology by acting as judges for the category winners in their sections. All judges were blinded to the identity of the entrants and the winners were selected based on the assessment of the photographs alone. Over 200 images were submitted from all over the world, giving us a good taste of the global diversity that exists not only in ecology, but in ecologists themselves.

We hope that you enjoy these amazing images and get a chance to learn something new!

## Winning images

There were so many wonderful submissions that narrowing the field down to the winners was very difficult. Congratulations to our winner Mohamed Shebl and our runners-up, Dhritiman Das and Daniel Wisbech Carstensen. We thank you for giving us the opportunity to experience the things you saw by sharing with us your spectacular pictures and the stories behind them.

In addition to our overall winner, this year there was a tie for overall runner-up, a testament to the quality of the images we received. Everyone is to be commended for their contributions and we hope that the contributors enjoyed sharing their work with us. We certainly enjoyed looking at all the images. Thank you to everyone who participated in this competition. We hope that you are inspired to continue taking pictures like these ones and that you will want to share them with others. If the creativity, talent and enthusiasm showcased here represent the current state and future direction of ecological research, then the field of ecology is certainly in good hands.

### Overall winner

**Fig. 1:**
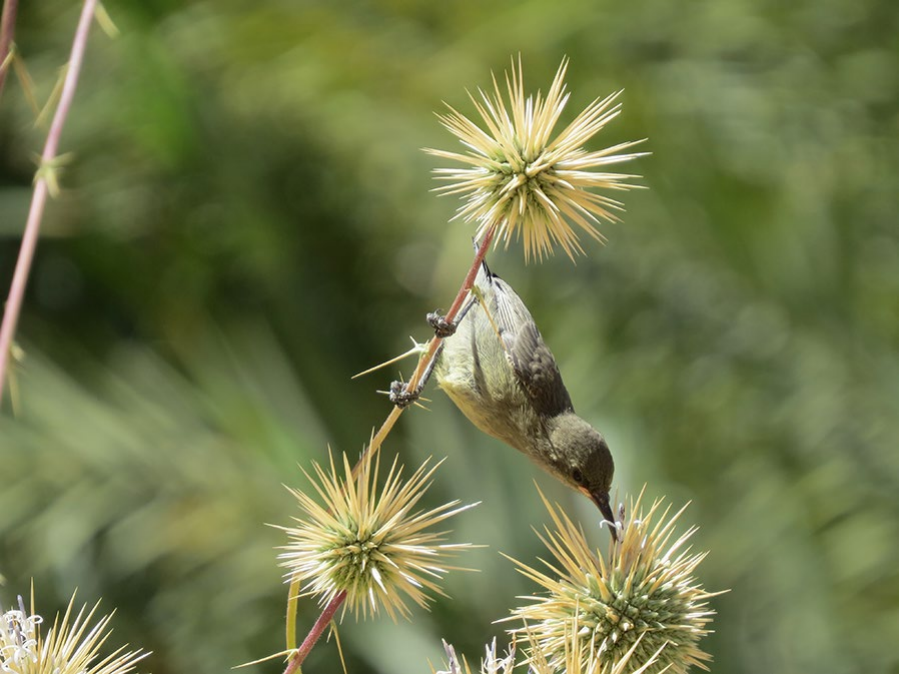
Overall winner. “Palestinian sunbird female forages on *Echinops* sp.” Attribution: Mohamed Shebl.

Mohamed Shebl (Department of Plant Protection, Suez Canal University Ismailia, Egypt) (Fig. 1):

The quiet beauty of this image is captivating; as this Palestinian sunbird reaches down to get some nectar from a rather intense looking plant (*Echinops* sp). Our guest judge Ana Porzecanski was very impressed: “*This is a truly gorgeous image and a great description of species from a part of the world that is underappreciated in terms of its biodiversity.*”

Indeed, this Middle Eastern bird reminds us that dense tropical rain forests are not the only places with rich biodiversity and highly adapted species. Mohamed Shebl tells us:

“This photo was taken at Deisa Valley, Tabouk, North West Saudi Arabia in April 2015. The bird is known from the Middle East and Sub-Saharan Africa. The male of the bird is completely different in color than the female; the male has a blue color. The bird has a long tongue and mostly feeds on the flower nectar.”

Navigating the spikes on those flowers is clearly a tricky feat, a task for which the bird is well-adapted. She is perfectly in tune with her environment and is enjoying a peaceful, and probably delicious, snack. The flashily colored male birds and their dramatic plumage are surely a sight to behold, but this image proves that you don’t have to be bright blue to attract admiration.

### Tie for overall runner-up

**Fig. 2:**
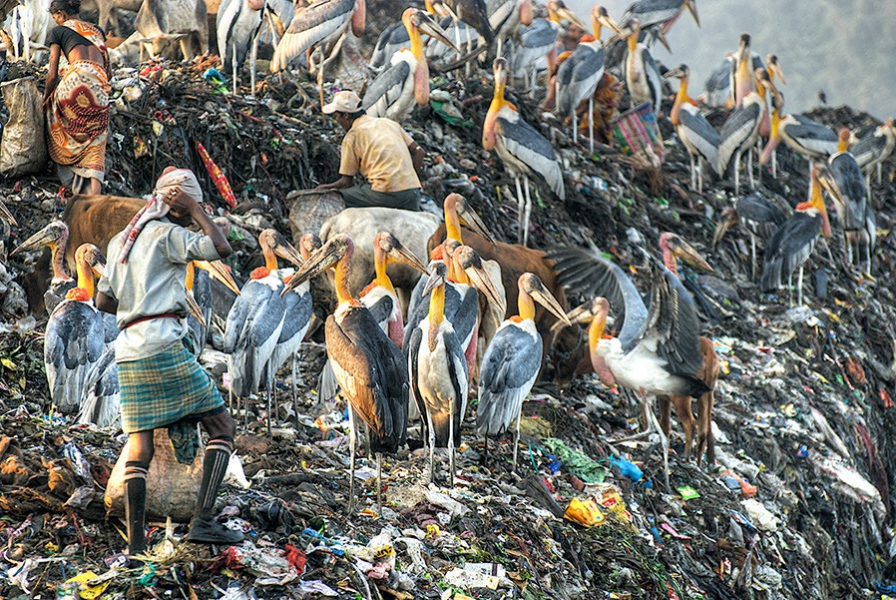
Tie for overall runner-up. “Greater Adjutant Stork (*Leptoptilos dubius*) foraging.” Attribution: Dhritiman Das.

Dhritiman Das (Ashoka Trust for Research in Ecology and the Environment (ATREE), India) (Fig. 2):

When natural habitats are destroyed by encroaching civilization, the displaced wildlife must search for new homes or otherwise perish. This striking image is an example of the intimate interactions that arise due to habitat destruction, where the severely endangered greater adjutant stork forages for sustenance in huge garbage piles of human refuse. Human activity is responsible for both the cause of and the cure for the plight of these storks. In this image, they appear untroubled by their close proximity to the people. This speaks to the adaptability of these amazing creatures and serves as a hauntingly prophetic message of the future. If more and more habitats are decimated, human-wildlife interactions will become more frequent and extensive with more fluid boundaries between “nature” and “developed areas”. Versatile species will be able to utilize makeshift refuges such as this one, while inevitably there will be some species unable to adapt. The storks show uncanny ability to thrive in an unnatural environment, but almost certainly would prefer their native wetlands to a garbage dump.

“The Greater Adjutant Stork (Leptoptilos dubius) is the world’s most endangered stork species with a total population estimated between 1,200 to 1,800 individuals. The Brahmaputra Valley is considered the last stronghold of this endangered stork and harbors more than 80 per cent of the global population of the species. Guwahati City’s urban garbage dumps now have the largest concentration of Greater Adjutant Storks in the world, because of ongoing destruction of the surrounding wetlands and habitat. These birds concentrate in urban disposal sites for foraging.”

### Tie for overall runner-up

**Fig. 3:**
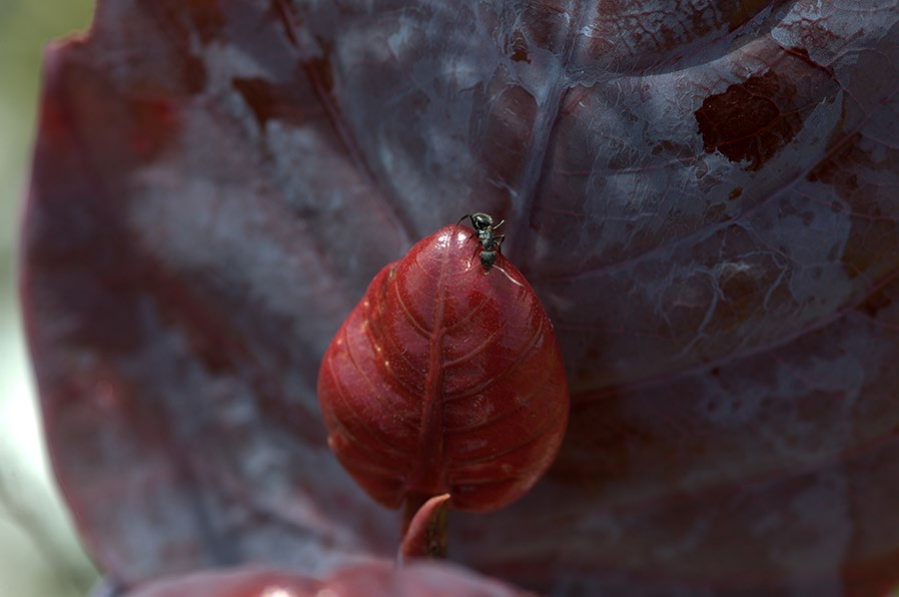
Tie for overall runner-up. “Camponotus ant patrolling a young leaf of the plant *Coccoloba cereifera*.” Attribution: Daniel Wisbech Carstensen.

Daniel Wisbech Carstensen (Department of Botany, BI-UNESP, Brazil) (Fig. 3):

This charming image, taken at Cipó National Park in Brazil, captures the mutually beneficial relationship between a carpenter ant and the very rare endemic plant *Coccoloba cereifera*, beautifully illustrating mechanisms of inter-species co-operation and highlighting how unique interactions can contribute to a location’s specific ecology. The succulent red leaf that the ant is drawn to exudes nutrient-rich nectar, providing nourishment for the intrepid ant, whose presence protects the vulnerable plant from herbivorous predators. Ant and plant both benefit from this symbiosis. As *C. cereifera* has extremely limited geographic distribution, this two-way transaction becomes even more fascinating and precious to study, existing nowhere else in the world. This image serves as a record of a unique relationship. How many more locally specific interactions remain under-characterized or undiscovered, and how will the rapid loss of biodiversity and habitat impact such singular relationships and our ability to learn about them?

“The young leaves have extrafloral nectaries, attracting the ants who in turn defend the leaves from herbivores. C. cereifera has a known natural distribution of only 26 km^2^ and is endemic to the Serra do Cipó national park in South East Brazil.”

## Section winners

### Behavioral and physiological ecology

**Fig. 4:**
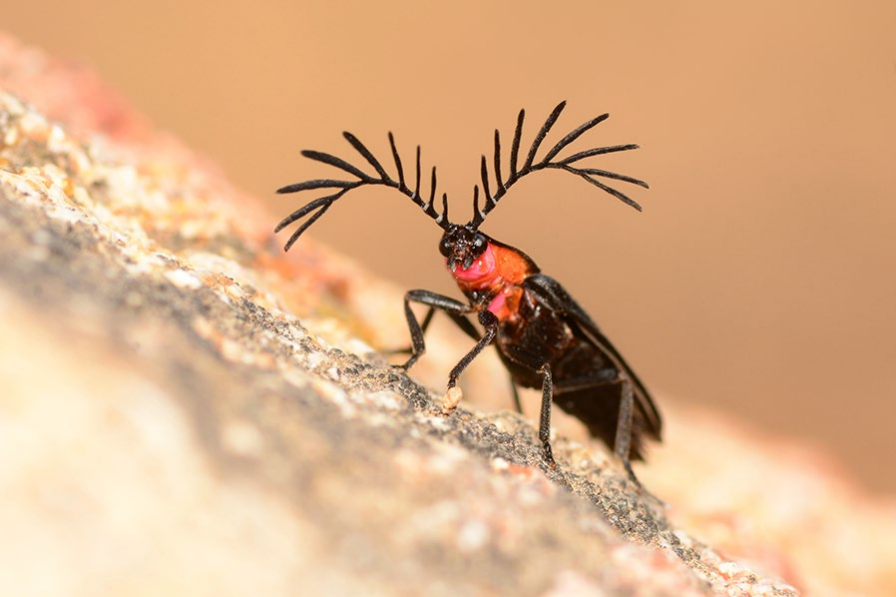
Winner: *Behavioral and Physiological Ecology*. “This beetle is a lampýridae from central Chile, and has some impressive antenna wish he uses to smell females.” Attribution: Bernardo Segura.

The popular *Behavioral and Physiological Ecology* section had a variety of entries that depicted some of the weird and wonderful attributes and actions of a broad range of organisms, some of which will have you believing that the ingenuity of evolution is limitless. Our winner in this category from last year has repeated the feat: Bernardo Segura from the Universidad de Chile once again has submitted the winning image for this section. This time, it is of a quite distinct beetle (Fig. 4), whose remarkable headgear is attuned for a purpose much greater than merely aesthetics:

“This beetle is a lampýridae from central Chile, and has some impressive antenna which he uses to smell females.”

The signaling of one mate to another is an essential part of successfully perpetuating a species, and the amazing adaptation of this beetle gives us a sense of the lengths that nature goes to accomplish this all-important function. Section Editor David Hughes agrees, finding this image very compelling from a behavioral ecologist’s perspective:

“I like this as it signals the many modes of communication we can see in behavioral ecology.”

### Community, population and macroecology

**Fig. 5:**
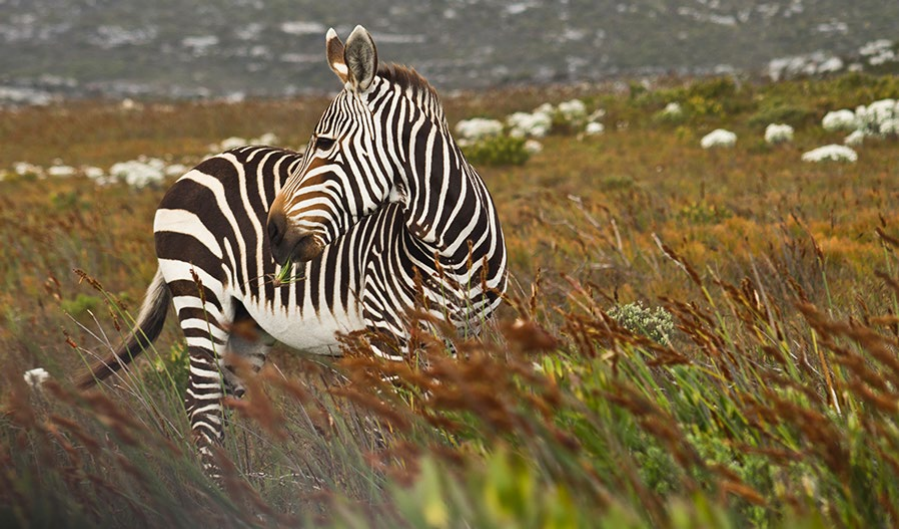
Winner: *Community, Population and Macroecology*. “This picture of a grazing cape mountain zebra (*Equus zebra zebra*) was taken near the Cape of Good Hope in South Africa. Cape Mountain zebra feed mainly on grass, the red grass *Themeda triandra* and other climax grasses such as finger grass *Digitaria eriantha* and terpentine grass *Cymbopogon plurinodis* being particularly favored.” Attribution: Julia Spaet.

The *Community, Population and Macroecology* section featured a range of pictures of multicomponent environments, highlighting the deep integration that members of the same ecosystem have. Julia Spaet, from the Red Sea Research Center, King Abdullah University of Science and Technology, Saudi Arabia submitted her winning image taken near the Cape of Good Hope, South Africa of a zebra (*Equus zebra zebra*) grazing in the African grasslands (Fig. 5). The relationship between the zebra, the grass and the climate is highly interconnected, and the preferences of the zebra are dictated by the larger environment, as Julie tells us:

“The height of grass is important—the zebras favor grasses between 50 and 150 mm in length. They will accept forbs and dwarf shrubs, but only during dry periods when grass is scarce.”

Zebra makes adjustments to their diet depending on the seasonal availability of the favored grasses. This beautiful image is an effective illustration of an animal responding to its dynamic environment, contributing to maintaining the balance of accessible resources.

### Conservation ecology and biodiversity research

**Fig. 6:**
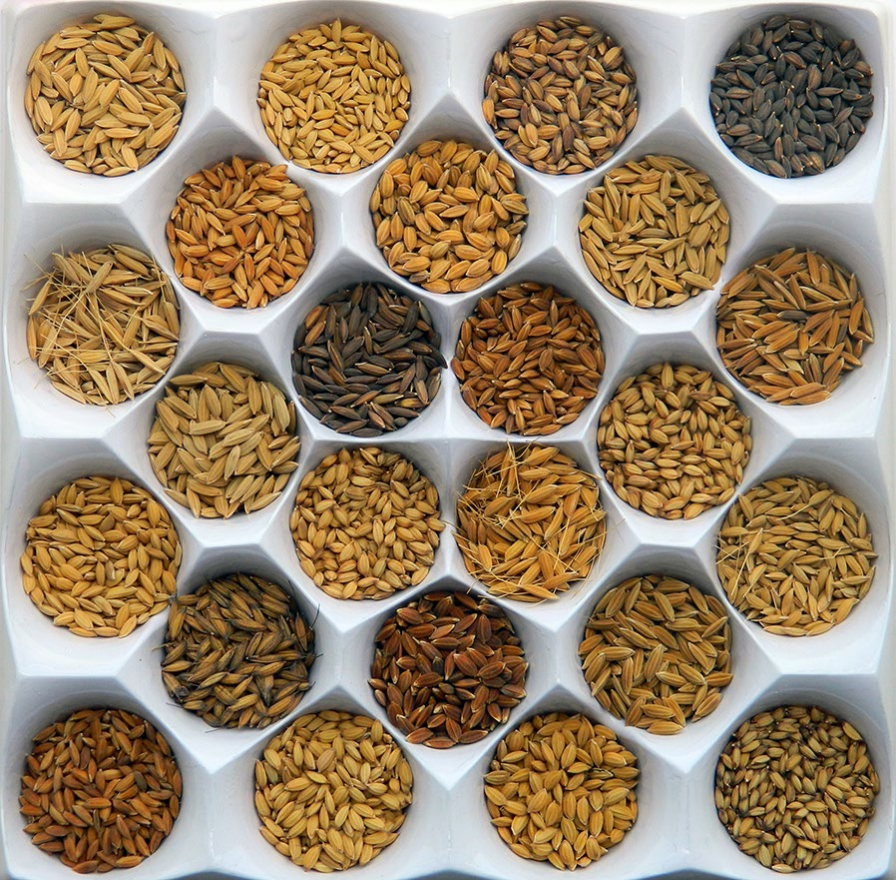
Winner: *Conservation Ecology and Biodiversity Research*. “Rice, the staple food crop of millions, contributes a large part of the caloric intake of people. India possesses enormous wealth of diversity in rice, especially for non-Basmati type, short grain aromatic rice. This photograph shows grain diversity, in both grain shape/size and lemma-palea color, revealing a diverse set of short grain aromatic rice collection for their utilization.” Attribution: Pritesh S. Roy.

The *Conservation Ecology and Biodiversity Research* section was our most popular category in 2015, representing over a third of all entries. This is a testament to the recognition of the fragility of our environment and the importance of preserving it in the face of a rapidly changing world. Rich resources of biodiversity buffer against these changes, and Pritesh S. Roy from the Central Rice Research Institute, India submitted a striking example of intrinsic diversity with his winning image of different varieties of rice (Fig. 6). He emphasizes the worth of this store and worries about its potential loss:

“These valuable heritage resources are on the verge of extinction due to a lack of research attention. However, these exotic germplasm possess many novel traits and genes which can be efficiently utilized in various crop improvement programs.”

Section Editor Josef Settele thought that this image was an important reminder to conservationists that natural variation is some of our most precious agricultural treasures:

“Many researchers in the field of conservation tend to ignore the biodiversity which was “created” by human use. The long history of rice cultivation has led to a huge diversity of varieties and cultivars which offer enormous potential for adaptation of agricultural systems to changing environments (including. climate change). These are alternatives to a technology-driven development in the agricultural sector and deserves much more attention by ecologists, as it is food security which should concern us most in order to prevent further losses of other highly diverse (semi-)natural areas. The sustainable use of traditional knowledge and thus culturally developed biodiversity is key to the conservation of biodiversity in general.”

### Landscape ecology and ecosystems

**Fig. 7:**
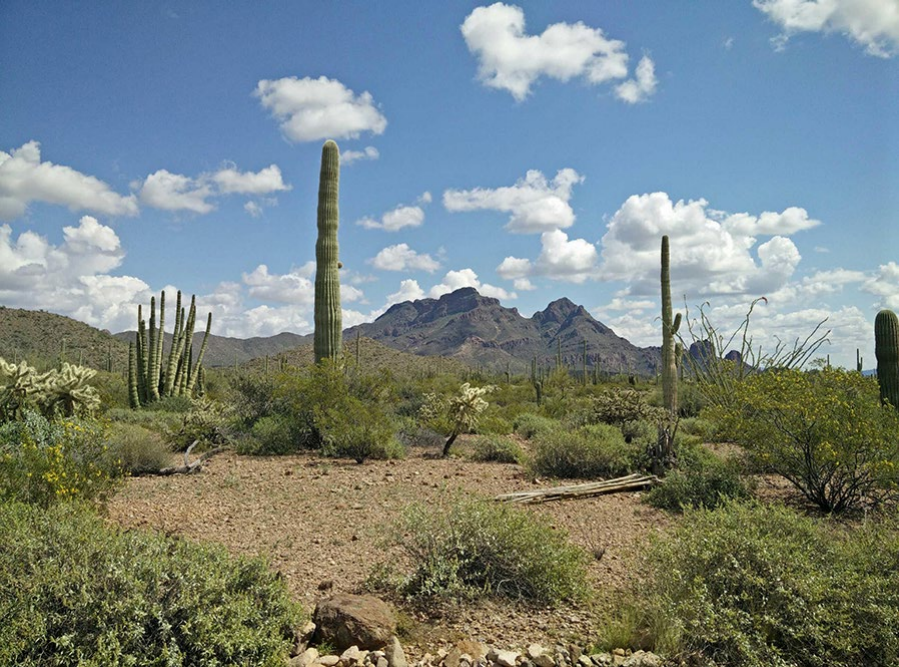
Winner: *Landscape Ecology and Ecosystems*. “Deserts are alive. The scene pictured captures a time of year when the Sonoran desert in North America becomes an oasis: the winter growing season. Here in Organ Pipe Cactus National Monument in southern Arizona, winter rains, relatively cool temperatures, and overcast skies allow annual plant species to germinate and perennial species to flourish, creating suitable habitat and food for migratory species and year-round inhabitants.” Attribution: David Winkler.

Some of our most breathtaking entries were to the *Landscape Ecology and Ecosystems* section, where the majesty of the land was highlighted and celebrated. David Winkler of the Department of Ecology and Evolutionary Biology at the University of California, Irvine certainly succeeded in capturing the essence of a desert ecosystem (Fig. 7). As David explains:

“Deserts are alive. Deserts are diverse and hospitable to millions of organisms around the world. “Extreme” landscapes and the stigma of barrenness associated with deserts are misnomers. Evolution acts in deserts as it does anywhere else on planet Earth, perhaps even more so. The “barrenness” of the desert brings with it perhaps one slightly obvious advantage: geological processes and features of the landscape are visible as far as the eye can see. Accounting for nearly 1/3 of the Earth’s land surface, deserts are not to be underestimated or undervalued. Deserts are alive.”

Section Editor Michel Baguette couldn’t help but praise this wonderful image:

“Like last year, it’s a picture of a desert. But this year we have these very nice cacti! Such columnar cactus are pollinated nightly by bats (the lesser long-nosed bats, Leptonycteris curasoae), which pollinate the flowers by feeding on their nectar. Later in the year, the bats become fruit-eater and eat the fruits of the cactus. Doing so, they participate to the dispersal of the seeds. This nice interaction between the cactus and the bats, shows the power of co-evolution.”

### Theoretical ecology and models

**Fig. 8:**
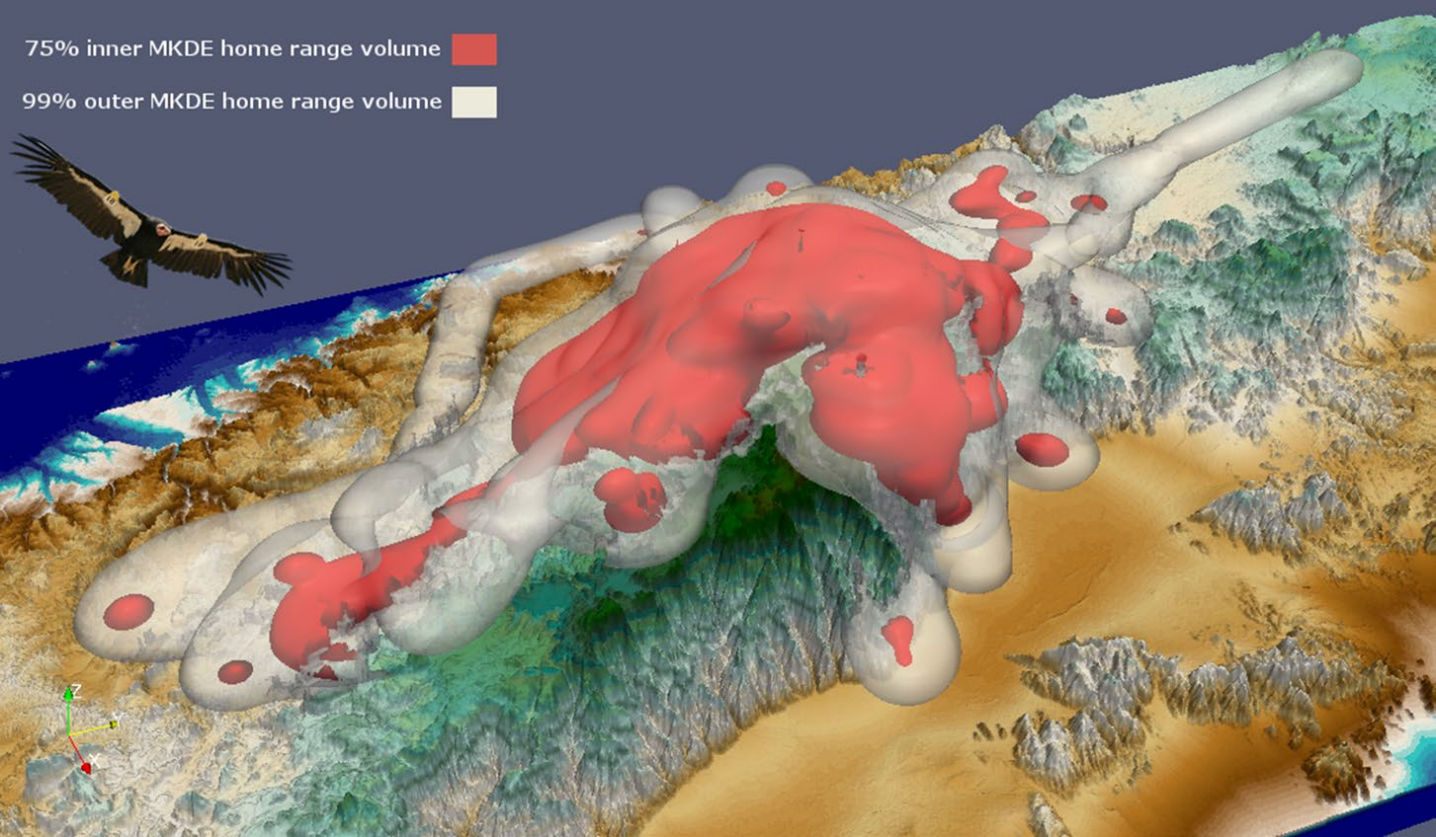
Winner: *Theoretical Ecology and Models*. “3D home range model calculated using GPS tracking data acquired from a wild California condor. This figure demonstrates 3D MKDE home range volumes calculated from GPS tracking data acquired from a free-ranging California condor reintroduced to the mountains of Baja California, Mexico. The red 3D volumes indicate the inner core 75% home range used by this bird and the white 3D volumes indicate outer regions used less often. The home range volumes were generated using a 3D terrain model of the mountains as a lower bounding layer.” Attribution: James K. Sheppard.

Creativity always goes into the entries to our *Theoretical Ecology and Models* category, which feature examples of the complex tools utilized by ecologists to model behaviors and environments. This year, James K. Sheppard from the San Diego Zoo Institute for Conservation Research provided this image of tracking data obtained from California condors (*Gymnogyps californianus*) that were re-introduced into the wild (Fig. 8). James tells us how technological advances have helped improve estimating wildlife location and range:

“3D home range model calculated using GPS tracking data acquired from a wild California condor. Miniaturized telemetry devices, such as those incorporating high-resolution GPS, have been used to remotely track free-ranging wildlife for decades. Biologists often summarize their tracking datasets using a home range estimator that describes the probability of an animal location at an arbitrary time during which the animal was observed. Modern telemetry devices acquire 3D location data in x and y planar geographic coordinates (e.g. latitude and longitude) as well as the z vertical dimension (e.g. altitude for flying animals or depth for swimming animals). However, analyses of animal space and habitat use have typically been 2D only, with the z-dimension examined separately or simply ignored. Disregarding the vertical component may seriously limit understanding of animal habitat use and niche separation. San Diego Zoo Global, USGS and the San Diego Supercomputer Center collaborated on developing a novel 3D movement-based kernel density estimator (MKDE) of animal home ranges.”

Models like these will become increasingly important to conservation efforts in the future, as James explains:

“Analyses and visualization using 3D MKDEs can be more informative and yield greater ecological insights than traditional 2D home range estimators in representing the space use of animals that have a substantive vertical component. Improved ecological understanding derived from our 3D method can in turn enhance the conservation management of endangered species. For example, we are using these 3D home ranges to more accurately model condor spatial patterns in relation to the increasing threat posed by collisions with wind farm turbines.”

### Editor’s pick

**Fig. 9:**
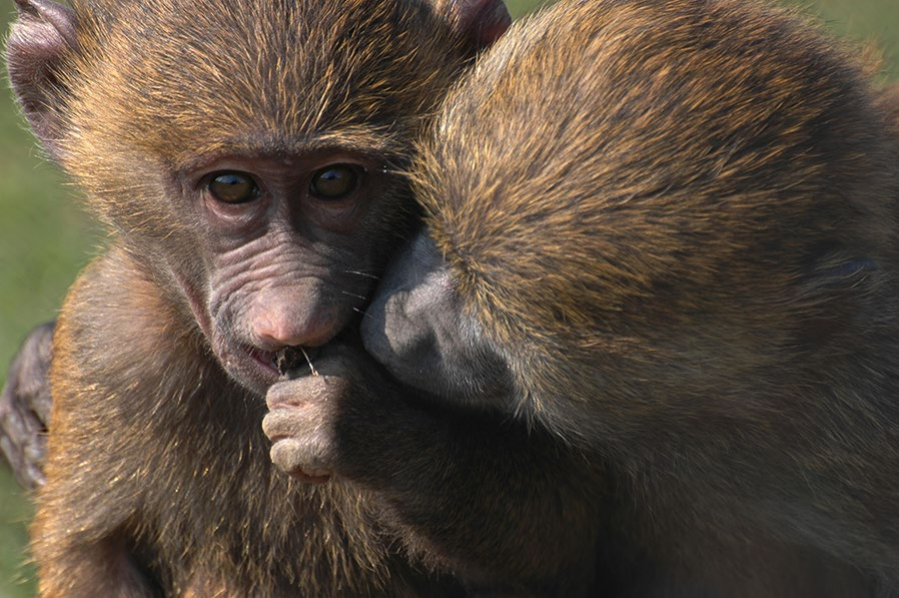
Winner: *Editor’s Pick*. “As one juvenile baboon (*Papio* sp.) eats, another inspects the food item.” Attribution: Catherine Markham.

Ecology is the science of interactions, and in the vast web of life, no agent exists independently; everything is connected. Catherine Markham from the Department of Anthropology at Stony Brook University, New York, has captured an especially intimate and poignant connection with her striking image of shared moment between a pair of juvenile baboons (*Papio* sp.) (Fig. 9). A close social network is important to many species and contributes to both fitness and survival, as Catherine’s research focuses on:

“As a primate behavioral ecologist, I’m interested in how the social bonds between individuals affect foraging strategies and success and—ultimately—individual survival and reproduction.”

We look on this picture with immediate familiarity, recognizing that the tenderness displayed by the baboons has counterparts in human behavior. So, just as the baboons have a connection to each other, they serve as a reminder that they also have links to us.

### Highly commended

There were so many fantastic submissions this year and we were astonished by the extraordinary combination of science and artistry that the photographs represented. Therefore, we wanted to share some more of the outstanding images we received to highlight the talent of our entrants as well as to showcase a vast range of ecological science.

Here are some of our favorites, organized by various ecological themes that emerged while looking through the entries. Behind every photograph is a story, and there are some incredible ones here.

#### Co-operation and harmony

Fundamentally, the world can be perceived as one big compromise, with bargains and trade-offs defining relationships between organisms across all ecosystems. Establishing harmonious relationships with the surrounding environment is not only a smart life strategy, but a driving force of inter-dependence, adaptation and evolution.

A fine example of a mutually beneficial interaction, as we have already seen in a runner-up image, can also be found in this image of Weaver ants (*Oecophylla smaragdina*) poised on top of a Lycaenidae caterpillar (Additional file 1). Although it looks like the ants are swarming to feed on the defenseless caterpillar, they are actually eating nutrients produced by the caterpillar while at the same time acting as a living shield to protect against predators. This is an excellent portrayal of a co-operative interaction that benefits both species expertly captured in this remarkable image by K. Vineeth Kumar from Mangalore University.

In a different example of co-operation, an “unlikely association” of a hippopotamus and a buffalo peacefully relaxes in the same waterhole (Additional file 2). Says Graeme Shannon, from the Mammalian Behavior and Evolution group at the University of Liverpool: “*Neither species are well known for their tolerance but rather for being bad*-*tempered and aggressive. After more than a decade of conducting savanna fieldwork, this is the first time I have seen a buffalo and hippo in such close proximity to one another*”. It is truly amazing to see these creatures sharing this limited resource so placidly, and how lucky for us that Graeme was there to record it.

As notable as inter-species tolerance is, the harmony between members of the same species is also something to highlight, as these playful Asiatic black bears (*Ursus thibetanus*) enjoy a sunny winter day in their natural environment (Additional file 3). Forging robust social bonds is a very successful evolutionary strategy and for some species the strength of the community network directly relates to the health and survival of the individual. We get a glimpse of that connection in this delightful image taken by Kainaat William from the Bioresource Research Centre in Pakistan.

#### Everywhere a threat

Not everything, of course, is a paradise of peace and co-operation. We had many entries that depicted a threat to the life of an organism, whether that harm came from animals, plants, fungi or the elements, reminding us that antagonistic interactions abound, and it is a dangerous world out there.

The image of this Ecuadorian amblypygi (*Heterophrynus batesii*) feeding on the giant golden silk orb-weaving spider (*Nephila* sp.) is truly frightening (Additional file 4). This is especially true considering that, according to Kenneth J. Chapin from the Department of Ecology and Evolutionary Biology at the University of California, Los Angeles, “*the specimen pictured is larger than the human hand*”. This is a visceral display of predator–prey interaction, skillfully captured for our amazement.

For a different perspective on predation, Harisoa Rakotonoely from the Graduate school of Environmental Science at Hokkaido University, Japan, shares this stunning image of an ant being trapped by the carnivorous plant *Drosera rotundifolia* (Additional file 5). Due to their immobility, plants need to employ elaborate mechanisms to attract and successfully capture prey, as Harisao describes: “*The more this ant moved, the more it got entangled and stuck until it became completely enveloped in the trap*”. This thrilling image reminds us that carnivorous diets are not restricted to the realm of animals and that the plant kingdom has its share of predators as well.

Parasitism represents another type of antagonistic interaction commonly found throughout the biosphere. This bizarre image of an *Ophiocordyceps* fungus attaching itself to the trap-jaw ant (*Odontomachus hastatus*) shows a host-parasite interaction with a particular twist (Additional file 6). According to João Araújo from the Pennsylvania State University, “*This fungus has the ability to change the ant’s behavior, making the poor insect leave the nest to die on the mossy base of trees*”. The parasite, then, does not merely have consequences for the host, but also affects other interactions in the community by changing the surrounding environment.

Moving from the animal kingdom, hostile environments also are encountered constantly, and can be threats that all too often prove to be fatal. A specific example of this is provided by Christina Giordano from the Department of Ecology and Evolution, Stony Brook University, who studies sea turtles off the coast of Long Island, NY, USA (Additional file 7). As she states: “*This photo, captured at Wantagh Park on Long Island, NY, depicts how the ecosystem that is home to sea turtles in the summer months is altered with the change of seasons. This landscape is a potential stranding site for sea turtles that do not migrate down south before the change of season. As Long Island waters cool, sea turtles begin their migration. Those that do not make this migration in time are subjected to freezing conditions, depicted above, and become cold*-*stunned.*” Some turtles do not survive this cold-stunned state, making the cold a threat that is just as serious to the turtles as any swimming predator. This image depicts a danger that is also quite beautiful and reinforces the idea that it is a constant endeavor to survive when environmental conditions change throughout the year.

#### Need for protection

Sometimes the greatest threat of all is posed by humanity itself. Whether by poaching or pollution, human activity has an enormous impact on the environment and all its inhabitants.

Being valued for fur, fins, horns or skin can be a hazardous condition. Although this image of an oriental rat snake (*Ptyas mucosa*) conveys a subtle menace, it is in fact the snake that is in peril (Additional file 8). As Mark Auliya from the Department of Conservation Biology at the Helmholtz Centre for Environmental Research, GmbH-UFZ, states: “*Since the early 20th century, the species is involved in the international skin industry. In 1990 the species was listed on Appendix II of CITES (Convention on International Trade in Endangered Species of Fauna and Flora) that regulates trade by an export permit, which is granted through the relevant management authorities. Particularly populations on Java (Indonesia) are intensively harvested for their skins. The 2015 export quota from Indonesia is 89,559 skins and skin products plus 441 live specimens*”. Quotas are a first step in responsibility, but they do not necessarily guarantee the protection of these creatures. The proximity of the snake to the person is nicely demonstrated in this image, silently emphasizing even more the vulnerability of this highly sought-after animal.

Infectious diseases are an ever-present threat that can have devastating consequences susceptible species, with human activity often contributing to the severity and spread of outbreaks. Amphibians are subject to the fungal disease chytridiomycosis, which is highly infectious and has caused massive decline in amphibian populations, especially in Central America. Anthropogenic effects accelerate the spread of the fungus, and this image of a frog being held by a gloved hand from Mark Spangler of the University of Alaska illustrates the precautions taken by ecologists doing field work to ensure that they do not adversely affect anything in the sites which they are studying (Additional file 9). Says Mark: “*Among those who work with amphibians first*-*hand, extreme care must be taken not only to prevent harming delicate individuals, but also to prevent any possibility of spreading the disease among individuals or across habitats*”. This simple image shows how those on the frontlines of ecology research protect their subjects in more ways than one, suggesting perhaps, that much like charity, conservation begins at home.

The zero-sum game of natural ecosystems versus human development generally results in ever-increasing habitat destruction, forcing species to either adapt or perish. Horseshoe crabs (*Tachypleus tridentatus*) live in beautiful, increasingly more developed coastal areas but they remain an unprotected species due to the lack of data about their populations and ecology. To help remedy this, Billy Kit Yue Kwan from the City University of Hong Kong studies the movement, habits and range of juvenile horseshoe crabs. He submitted this evocative image of horseshoe crabs in the midst of a highly developed area in Hong Kong (Additional file 10). He tells us: “*The picture was taken during a research on the home range area utilized by the juveniles on an intertidal mudflat in Hong Kong, in which the shore is opposite to largely reclaimed Hong Kong International Airport and the newly developed Tung Chung Town, separated by a narrow waterway. On top of the shore is the Ngong Ping Cable Car connecting Tung Chung Town and the famous tourist attractions, Po Lin Monastery and the Tian Tan Buddha*”. Possible designation of this as a conservation area in the future depends on the collection of these data, emphasizing the importance that ecological research can have in helping to preserve both environments and populations. The juxtaposition in this image of the verdant mountain, beautiful waterway and thriving horseshoe crabs with the cable car tracks and high-rise hotels acutely underlines the degree to which natural landscapes are losing ground to the juggernaut of civilization.

Sometimes the loss of one species can lead to the loss of others. This image by Mohd Masri bin Saranum from the Malaysian Agricultural Research and Development Institute shows a stingless bee determinedly barreling towards a flower that is ripe for pollination (Additional file 11). This is a critical process that is crucial to floral reproduction and diversity and that is dependent on the bees, who themselves are under threat. Mohd Masri wisely states: “*With the number of bees in decline because of pesticide and colony collapse disorder (ccd), the need for conservation is getting more and more important*”. Loss of bees will lead to loss of flowers and will have further chain-reaction consequences both foreseen and unknown. Conservation efforts centered on bees, then, will have far-reaching effects on many other members of the ecosystem. Looking at this outstanding image, it is easy to understand why we should be motivated to preserve and save these magnificent organisms and, by extension, their surrounding ecosystems.

#### Awesome adaptations

There were many images that featured extraordinary examples of the adaptations that make organisms precisely suited for their respective environments. How something looks or acts can give us a lot of information about an organism, including where it lives and what it eats. Through studying adaptations, we can also deduce the evolutionary forces that selected for specific behaviors or traits, giving us deeper insight into the ecology of species.

Himalayan blue sheep (*Pseudois nayaur*) can thrive at high altitudes because they have adapted to the low oxygen availability and they are hidden from predators by their camouflage (Additional file 12). This image was taken at a height of 5600 meters above sea level by Kumar Singh from the University of Delhi, who says that the sheep “*are exceptional in climbing high mountains and show excellent camouflage capability with the surrounding environment.* P. nayur *remain in large groups and shows aggressive territorial behavior and the species play a crucial role in ecology of the surrounding nutrient poor* *habitat*”. We are shown by this picture how the sheep blend into the landscape, and we can imagine them hiding from the sharp eyes of the snow leopard (*Uncia uncia*), the area’s top predator.

In South Africa, long-tongue flies (*Prosoeca ganglbaueri*) have co-evolved with the floral nectar tubes from which they feed, and nudged along by an elongation arms race with the flowers, their proboscis can grow up to 50 mm in length (Additional file 13). Michael Whitehead of Australian National University submitted this image and tells us that “*this specialist nectar*-*feeder is now the exclusive pollinator for over 20 species of long*-*tubed flower. This however does not stop it from nectar robbing of shorter*-*tubed species such as the Agapanthus seen here. Given the remarkable diversity of plants that have come to rely on them as sole pollinator, the long*-*tongue flies of Southern Africa are truly an exemplar keystone species*”. This gorgeous image’s depiction of an extraordinary adaptation lets us see an extreme example of what can happen when evolutionary forces take control and lets us play an intellectual game to ponder what the upper limit of fly proboscis length could be.

The ability of the greater bamboo lemur (*Prolemur simus*) to eat large amounts of cyanide-containing Madagascar giant bamboo (*Cathariostachys madagascariensis*) gives it an advantage over competitors, allowing it to have a monopoly on an entire food source that is unavailable to others (Additional file 14). As Peggy Boone from the University of Wisconsin-Madison describes, “*On average, this lemur daily consumes ten times the amount of cyanide that would be lethal to other mammals of similar size. While it is not yet known how this primate is able to safely ingest the toxins, its physiological adaptations have allowed for the utilization of a widely available resource with little competition from other species*”. We see the lemur’s curious face peering out from the forest, and we can wonder at the physiological adaptations that enable it to survive and flourish in this particular ecosystem.

#### Parental interactions

The definition of evolutionary success is reproducing and passing on genetic traits to the next generation. Unsurprisingly, then, we had many images that depicted particular interactions between parents and their progeny.

This image submitted by Alma Rosa Moreno Pérez from the Instituto de Ecología at Ciudad Universitaria, Mexico exemplifies the extraordinary lengths that parents will go to for their offspring (Additional file 15). Says Alma: “*This is a pregnant* Leptonycteris yerbabuenae*. This female carried her baby in her womb from the coast of Jalisco to the Sonoran desert, where, it met another 100,000 to 300,000 females to give birth in a maternity cave in the Biosphere Reserve of Pinacate and Great Altar Desert in Sonora, Mexico*”. Viewing this image, one can almost feel the weighted burden that this mother carries along with her as she makes her long journey to safely deliver her young.

Parents also are teachers, responsible for raising self-sufficient progeny, who often learn through mimicry, charmingly illustrated by this submission of Michelle Achlatis from the University of Queensland, taken on Heron Island in the Great Barrier Reef (Additional file 16). The noddy tern chick looks like it is trying to emulate its proud parent, whose behavior will be the template for the chick’s education. Learning how to avoid danger is one of the most important lessons, and Michelle explains why this is vital for life in a Pisonia tree (*Pisonia grandis*) nest: “*The sticky fruits from the tree are a deadly trap for the unwary youngsters or even the adult noddies. Death by immobilization is so common on the forest floor that it is hypothesized as an evolutionary strategy of the tree for fertilizing the soil with decaying animal matter*”. No mother would want her chick to succumb to this mire of Pisonia fruit, so it is encouraging to see this little one already taking cues from its wiser, more experienced mom.

Offspring require an incredible investment of energy and resources and they need to be especially protected before they are born. Therefore, having a partner around to share the burden can be really useful. Ravichandra Mondreti from Pondicherry University provides an image of two sooty terns both caring for their egg (Additional file 17). As Ravichandra explains: “*Here the pair is seen taking turns incubating and shading their only egg. The egg would be incubated for approximately a period of 29* *days. During this period the bird is attentive over 95% of the time incubating when it is cool and shading when it is hot*”. The partnership and co-operation of the mother and father is crucial to the survival of the egg, and the grueling incubating schedule could not be handled by only one individual. In contrast, the mother euryhaline Mozambique tilapia (*Oreochromis mossambicus*) can act as a solo agent, incubating fertilized eggs in her mouth for their protection (Additional file 18). This image, taken by Andre P. Seale from the University of Hawaii, starkly highlights the fragility of the young offspring in the face (literally) of the much larger mother and points to why parental protection developed as such a wide-spread adaptation.

#### Colorful diversity

Nature explodes with color and there were some striking submissions that captured the beauty and variety of hues found throughout different environments, from under the ocean to embedded in forests.

The deep, rich color of this intertidal seaweed was photographed by Levi Pompermayer Machado from the Universidade Federal do Espírito Santo (Additional file 19). He states: “*The ecological importance and fragility of macroalgae should be noted. However, it can develop into one of the most dynamic and complex ecosystems, providing a beautiful natural spectacle*”. The colors and textures in this image positively leap out at us, and we are enthralled by the fact that even algae can be beautiful.

Similarly, the many vibrant colors found in the cold water gardens of South Australia display the diversity of this community, while stunning us with their splendor (Additional file 20). Daniel Gorman from the Universidade de São Paulo took this image of “South Australia’s secret gardens” and explains, “*This image was taken beneath the Stenhouse Bay jetty on the rugged coastline of Yorke Peninsula, Australia. A beautiful and fragile basket star (*Astroboa ernae*) shares a pylon with a colony of blue ascidians (*Clavelina molluccensis*), sponges, brown and red algae as well as a multitude of other species*”. The myriad colors in this image reflect the level of biodiversity of the water garden and serves as a marker of the complexity of this underwater ecosystem.

Back on land, the intricate web of the tube-dwelling spider (*Ariadna* sp.) is found nestled in a cluster of bryophytes, which create dazzling mosaics (Additional file 21). Our runner up Bernardo Segura took this image at Altos de Cantilana in Chile and he tells us “*This place is a high diversity and high endemism area of bryophytes*”. That diversity is perfectly captured by this photo, along with the very elaborate spider web embedded in the plants.

This grove of baobab trees (*Adansonia grandidieri*) is surrounded by a pool of shallow water with bursts of vibrant purple lilies punctuating the landscape (Additional file 22). Kathryn M. Everson from the University of Alaska Museum describes her image: “*These trees are endemic to Madagascar and they form a striking landscape that draws travelers from around the world. The area is surrounded by stands of shallow water filled with lilies*”. The flowers stand out against the dark water, allowing us to marvel at their glorious color while reminding us that our earth is a precious treasure that should be cherished and preserved.

#### And finally…

We would like to leave you with this image of ecology research in action. This photograph of a brilliant-thighed poison frog (*Allobates femoralis*) father transporting tadpoles, wearing a tracking device was taken by Andrius Pašukonis from the Department of Cognitive Biology at the University of Vienna (Additional file 23). Says Andrius: “*We study the movement patterns (where they go) and the orientation mechanisms (how they find their way around) of poison frogs in the Amazon. We use miniature tracking devices to quantify the movement patterns of these small frogs in the field.* *Our findings reveal complex movement ecology and are indicative of a highly developed spatial learning ability in poison frogs. In this photograph, you see one of the male frogs being tracked at our field site in French Guiana while carrying his offspring to the water*”. We see the tracking device on the frog and immediately gain deeper insight into ecological research and the tools used to conduct it.

Section Editor David Hughes also shared his thoughts: “*This I really like as it shows how we behavioral ecologists are getting better.*”

This image competition certainly provides ample evidence that ecologists are already amazing, curious and talented individuals and that their hard work and enthusiasm contribute positively to education and conservation efforts. After all, we must all share this one home, so studying the depth and complexity of ecological interactions not only gives us information to help understand our world, but also to treasure it.

We hope that you have enjoyed viewing these images and that the beauty and wonder captured by them has inspired you to see the world in a new light, with increased awareness and appreciation.

### Additional files

Additional file 1: “As a field researcher I keep exploring nature. One day I was in search of Weaver ants (*Oecophylla smaragdina*) at the University garden, I was astonished to see something interesting happening. I found a few Weaver ants encircling a caterpillar. On close inspection we found that the ants were not actually feeding on the caterpillar, but in contrast they were protecting the caterpillar. Later we got to know that this is a symbiotic relationship between the Weaver ant and the caterpillar of a butterfly family named Lycaenidae (Blues).The Caterpillars of Family Lycaenidae are known to possess glands that secrete sugary substance that attract ants. Ants swarm over the caterpillar to extract the sugary substance from it and also they are known to tickle the caterpillar with their antennas for the same purpose. In return as a reward the Weaver ants provide protection to the Caterpillar from predatory species.” Attribution: K. Vineeth Kumar (Mangalore University).

Additional file 2: “While conducting research in the Masai Mara, Kenya on the formation of mixed species groups, we spotted a hippo and buffalo sharing a rather small mud wallow. Neither species are well known for their tolerance but rather for being bad-tempered and aggressive. Nevertheless, they seemed to have come to an understanding that allowed both animals to benefit from accessing the wallow. Interestingly our research was focused on exploring which herbivore species liked to associate together and how resource availability and predation drive these aggregations (e.g. wildebeest & zebra). After more than a decade of conducting savanna fieldwork this is the first time I have seen a buffalo and hippo in such close proximity to one another.” Attribution: Graeme Shannon (University of Liverpool).

Additional file 3: “The beauty of this picture reveals the playful and socially positive interaction between 3 Asiatic black bears (*Ursus thibetanus*) as they enjoy the warm heat of the sun in winter season. This picture also shows their natural habitat in which they have adjusted and are enjoying it. *Ursus thibetanus* are found in variety of forested areas and in Pakistan they are found in Himalayan region.” Attribution: Kainaat William (Bioresource Research Centre, Pakistan).

Additional file 4: “The Ecuadorian Amblypygi *Heterophrynus batesii* feeding on the giant golden silk orb-weaving spider *Nephila* sp. Amblypygi are distant relatives of spiders and scorpions outfitted with large front claws (pedipalps) and long, antenna-like front legs. The specimen pictured is larger than the human hand. While amblypygids do not produce silk, the giant orbweaver produces enormous webs with strong golden threads. This photo is the first example of amblypygi feeding on a spider, and represents intraguild predation in the community. This photo was captured at the Tiputini Biodiversity Station near Yasuni National Park in Amazonian Ecuador. Yasuni National Park is heralded as the most biodiverse place on terrestrial earth, but is threatened by pollution and development of the petroleum industry.” Attribution: Kenneth J. Chapin (University of California, Los Angeles).

Additional file 5: “The *Drosera rotundifolia* is a widespread carnivorous plant in the wetlands of Japan. This picture shows an ant entangled in the sticky tentacles of the hungry plant, taken in the summer of 2013 in the Sarobetsu mire, northern Hokkaido. The more this ant moved, the more it got entangled and stuck until it became completely enveloped in the trap. The Sarobetsu mire was a coal mining site in the past, but now it is considered vulnerable area and has been converted into a conservation and research site, also attracting tourists by the colorful flowers blooming in summer.” Attribution: Harisoa Rakotonoely (Hokkaido University).

Additional file 6: “*Ophiocordyceps* undescribed species infecting the trap-jaw ant *Odontomachus hastatus* in Central Brazilian Amazon. This fungus has the ability to change ant’s behavior, making the poor insect to leave the nest to die on the mossy base of trees. There, the humidity is always constant, ensuring a permanent water supply for the parasite.” Attribution: João Araújo (Pennsylvania State University).

Additional file 7: “My work aims to quantify sea turtle diet on Long Island, NY. This data is obtained by analyzing stomach contents from stranded sea turtles over a 30-year time frame. Many sea turtles strand due to a condition known as cold-stunning. During the winter months, Long Islanders are encouraged to walk the shorelines as winter approaches to check for cold-stunned sea turtles. This photo, captured at Wantagh Park on Long Island, NY, depicts how the ecosystem that is home to sea turtles in the summer months is altered with the change of seasons. This landscape is a potential stranding site for sea turtles that do not migrate down south before the change of season. As Long Island waters cool, sea turtles begin their migration. Those that do not make this migration in time are subjected to freezing conditions, depicted above, and become cold-stunned. If the sea turtle does not survive this condition, we quantify the stomach contents to analyze sea turtle diet in Long Island waters. This research not only provides insight into sea turtle diet, but also paints a picture of the ecosystem these species are present in. We can analyze which prey species are available in the surrounding ecosystem, as well as their population trends over time.” Attribution: Christina Giordano (Stony Brook University).

Additional file 8: “The Oriental Rat Snake (*Ptyas mucosa*) is distributed from central Asia to East and South East Asia. The diurnal active species mainly preys on frogs and rodents in open agro-ecosystems. The photo shows an adult specimen in search for food close to a paddy field worker in Central Java (Indonesia). Since the early 20th century, the species is involved in the international skin industry. In 1990 the species was listed on Appendix II of CITES (Convention on International Trade in Endangered Species of Fauna and Flora) that regulates trade by an export permit, which is granted through the relevant management authorities. Particularly populations on Java (Indonesia) are intensively harvested for their skins. The 2015 export quota from Indonesia is 89 559 skins and skin products plus 441 live specimens.” Attribution: Mark Auliya (Helmholtz Centre for Environmental Research GmbH-UFZ).

Additional file 9: “More than one-third of amphibians are threatened with extinction globally. In certain parts of the world, such as Central America, this number may reach as high as one-half. A major contributor to amphibian decline in these areas is a devastating disease known as chytridiomycosis. Chytridiomycosis is a fungal infection that is believed to cause death by disrupting an amphibian’s ability to regulate water, oxygen, and electrolytes through its skin. The disease is highly infectious and human activity has greatly increased the spread of the fungus, which now occurs worldwide. Although certain species exhibit relative immunity, the disease has been implicated in the catastrophic decline or complete extinction of no less than 200 species. In places hit hardest by chytridiomycosis the disease may eliminate over half of all species from an area. Remaining species experience declines of up to 80%. These crashes unfold in as little as six months, with recovery time estimated at approximately 15 years. Unique to amphibians, chytridiomycosis is the only disease known to cause such massive decline in species not otherwise at risk of extinction. Additionally, declines caused by chytridiomycosis are believed to represent the largest loss of biodiversity attributable to disease. Fortunately, the spread of the chytrid fungus can be predicted once it has struck, its presence can be detected with diligent monitoring, and amphibian populations can be brought into captivity in order to avoid the disease. Such efforts, however, are massive undertakings which require widespread collaborations among extremely dedicated conservationists in multiple fields of study. Among those who work with amphibians first-hand extreme care must be taken not only to prevent harming delicate individuals, but also to prevent any possibility of spreading the disease among individuals or across habitats.” Attribution: Mark Spangler (University of Alaska).

Additional file 10: “My research is focused on the ecology and conservation of an ancient marine arthropod, horseshoe crab *Tachypleus tridentatus* in which the oldest fossil could probably be dated back to Late Ordovician Period. Their populations were in dramatic declines in many Asian places due to habitat destructions for coastal developments and harvest pressure for biomedical applications and consumption. Because of the lack of scientific data, this species is listed as Data Deficient on the IUCN Red List of Threatened Species, thus the animal is not being protected by neither the international treaties nor the local law. The picture was taken during a research on the home range area utilized by the juveniles on an intertidal mudflat in Hong Kong, in which the shore is opposite to largely reclaimed Hong Kong International Airport and the newly developed Tung Chung Town, separated by a narrow waterway. On top of the shore is the Ngong Ping Cable Car connecting Tung Chung Town and the famous tourist attractions, Po Lin Monastery and the Tian Tan Buddha. During the study in summer 2014, the juveniles were labeled by a waterproof red-colored plastic tag and allowed to move for foraging and normal daily activities. Their recaptured locations were recorded for home range calculations. The aim of this study was to provide preliminary data on the movement patterns and space utilization of juvenile horseshoe crabs in the field, which may be useful for the possible designation of conservation area in the future. While there is hardly any proposal for the habitat protection for this animal group, Tung Chung New Town Extension Project has been proposed and the EIA study was on the public inspection stage when the home range study was still conducting. Who will win in this space-competing game? Should be the Humans, with no doubt.” Attribution: Billy Kit Yue Kwan (City University of Hong Kong).

Additional file 11: “Conservation of pollinators consists of bees and native bees in Malaysia. With the number of bees in decline because of pesticide and colony collapse disorder (ccd), the need for conservation is getting more and more important. Compared to the bees, stingless bees are another pollinator in Malaysia which has currently gained popularity in Malaysia. The stingless bees *Trigona* sp. is being utilized as pollinator and also for its honey.” Attribution: Mohd Masri bin Saranum (Malaysian Agricultural Research and Development Institute).

Additional file 12: “*Pseudois nayaur* (Himalayan Blue Sheep) is a goat antelope found in greater Himalayas. This photograph is taken in Valley surrounding the Gangetic glacier at a height of 5,600 meters above the sea level. They are preferred meal for the snow leopard (*Uncia uncia*).They are exceptional in climbing high mountains and show excellent camouflage capability with the surrounding environment. *P. nayur* remain in large groups and shows aggressive territorial behavior and the species play a crucial role in ecology of the surrounding nutrient poor habitat.” Attribution: Vineet Kumar Singh (University of Delhi).

Additional file 13: “The remarkable proboscis of South Africa’s long-tongue fly (*Prosoeca ganglbaueri*) can attain lengths of over 50 mm. Due to the co-evolutionary feedback that drives length in both fly tongues and floral nectar tubes, this specialist nectar-feeder is now the exclusive pollinator for over 20 species of long-tubed flower. This however does not stop it from nectar robbing of shorter-tubed species such as the Agapanthus seen here. Given the remarkable diversity of plants that have come to rely on them as sole pollinator, the long-tongue flies of Southern Africa are truly an exemplar keystone species.” Attribution: Michael Whitehead (Australian National University).

Additional file 14: “A greater bamboo lemur (Prolemur simus) feeds on bamboo leaves during a rainstorm in Kianjavato, Madagascar. Madagascar giant bamboo (*Cathariostachys madagascariensis*), the primary food source of this species, contains cyanogenic glycosides that are highly toxic to most mammals. On average, this lemur daily consumes ten times the amount of cyanide that would be lethal to other mammals of similar size. While it is not yet known how this primate is able to safely ingest the toxins, its physiological adaptations have allowed for the utilization of a widely available resource with little competition from other species. Greater bamboo lemurs are critically endangered and occupy only 1-4% of their original home range in southeastern Madagascar. Leading threats include habitat loss and fragmentation as a result of slash-and-burn agriculture, mining, and illegal logging.” Attribution: Peggy Boone (University of Wisconsin-Madison).

Additional file 15: “This is a pregnant *Leptonycteris yerbabuenae*. This female carried her baby in her womb from the coast of Jalisco to the Sonoran desert, where, it met another 100,000 to 300,000 females to give birth in a maternity cave in the Biosphere Reserve of Pinacate and Great Altar Desert in Sonora, Mexico. This bat was in the endangered species list in Mexico (NOM-059), and after almost 20 years of conservation efforts, scientific research and education to the communities, last year it left the list because its populations are stabilized or increased. Nowadays the people in the Laboratory for Terrestrial Vertebrates Conservation in the Ecology Institute (UNAM) are still monitoring this and other colonies to keep an eye on the species wellness.” Attribution: Alma Rosa Moreno Pérez (Ciudad Universitaria).

Additional file 16: “Heron Island, Great Barrier Reef-February 2015. A proud noddy tern chick and parent stand guard at their Pisonia tree nest on Heron Island, Great Barrier Reef, Australia. Known for their nodding behavior during their courtship dances, the black tern *Anous minutus* normally lays one egg each breeding season. But this chick is a latecomer from a second round of offspring after a heavy storm brought down many of the original nests made in the peak breeding season. Strong winds are not the only threat to these birds; other dangers lurk closer to home. *Pisonia grandis* forms a lush forest on the small coral cay, partly supported by the guano of the large bird population that it fosters by providing shelter and nest building material. Yet its sticky fruits are a deadly trap for the unwary youngsters or even the adult noddies. Death by immobilization is so common on the forest floor that it is hypothesized as an evolutionary strategy of the tree for fertilizing the soil with decaying animal matter. Is the onshore productivity of the forest linked to the productivity in the surrounding waters, which host a colorful forest of their own? The coral reef encircling the lagoon is what brings marine biologists like myself to the island for scientific research. But even if you spend most of your time underwater at Heron Island you are guaranteed to encounter the noddies, as there are about 80,000 of them on the small coral cay at any time. You would think the island should be renamed after them, and they would nod in approval.” Attribution: Michelle Achlatis (University of Queensland).

Additional file 17: “A pair of Sooty Terns incubating in the only known extant colony of seabirds in Pitti Island, Lakshadweep, India. Here the pair was seen taking turns to incubate and shade the only egg. The egg would be incubated for approximately for a period of 29 days. During this period the bird is attentive over 95% of the time incubating when it is cool and shading when it is hot.” Attribution: Ravichandra Mondreti (Centre for Ecology and Functional Evolution, CNRS).

Additional file 18: “Tilapia are widely used in aquaculture, and for research. The euryhaline Mozambique tilapia (*Oreochromis mossambicus*) has a remarkable capacity to adapt to environments ranging from fresh water to double-strength seawater. For this reason, the Mozambique tilapia is a good model to study osmoregulation and the effects of environmental salinity on growth and reproduction. The Mozambique tilapia is a mouth-brooding species, originally found in the estuarine areas of southeast Africa. The female incubates the fertilized eggs in its mouth. During this incubation period, the fertilized eggs develop into yolk sac fry, which after 5 to 8 days become free swimming, as the swim bladder develops. Following absorption of the yolk, they become juveniles and no longer seek shelter in the female’s mouth. The image depicts a brooding female Mozambique tilapia with a yolk sac fry resting on its lower lip.” Attribution: Andre P. Seale (University of Hawaii).

Additional file 19: “The development of research in diversity and biochemistry of seaweeds in intertidal areas is possible because of the wealth of shapes and colors and despite the simple thalloid organization of such bodies. It should be noted the ecological importance and fragility of macroalgae that however can develop into one of the most dynamic and complex ecosystems providing a beautiful natural spectacle.” Attribution: Levi Pompermayer Machado (Universidade Federal do Espírito Santo).

Additional file 20: Additional file 20: “South Australia’s secret gardens—this image was taken beneath the Stenhouse Bay jetty on the rugged coastline of Yorke Peninsula, Australia. A beautiful and fragile basket star (*Astroboa ernae*) shares a pylon with a colony of blue ascidians (*Clavelina molluccensis*), sponges, brown and red algae as well as a multitude of other species. Temperature marine habitats can be stunningly beautiful and support high levels of biodiversity. These cold water gardens like their coral reef equivalents are threatened by human development and climate change so an improved understanding their ecology is vital for conservation.” Attribution: Daniel Gorman (Universidade de São Paulo).

Additional file 21: “The spider’s garden. This image shows the web of a tube-dwelling spider (*Ariadna* sp.), in a marvelous, high diversity garden of bryophytes in Altos de Cantillana, Central Chile. This place is a high diversity and high endemism area of bryophytes.” Attribution: Bernardo Segura (Universidad de Chile).

Additional file 22: “Along a dirt road in western Madagascar stands a grove of baobab trees (*Adansonia grandidieri*) collectively known as baobab alley. These trees are endemic to Madagascar and they form a striking landscape that draws travelers from around the world. The area is surrounded by stands of shallow water filled with lilies, where local children collect fallen baobab fruits. Local people call the baobab tree ‘renala’ meaning ‘mother of the forest.’” Attribution: Kathryn M. Everson (University of Alaska Museum).

Additional file 23: “Father brilliant-thighed poison frog (*Allobates femoralis*) transporting his tadpoles while wearing a tracking device. Neotropical poison frogs (*Dendrobatidae*) show some of the most complex social and spatial behaviors among amphibians. In many species males shuttle tadpoles on their back from terrestrial territories to small ephemeral pools spread wide and far around the rain forest. We study the movement patterns (where they go) and the orientation mechanisms (how they find their way around) of poison frogs in the Amazon. We use miniature tracking devices to quantify the movement patterns of these small frogs in the field. Our findings are revealing a complex movement ecology and suggesting highly developed spatial learning ability in poison frogs. In this photograph, you see one of the male frogs being tracked at our field site in French Guiana while carrying his offspring to the water. The photograph actually captures the last moments of his life. Not long after I took this picture the frog was predated by a snake. Luckily, this happened right after he completed his parental duties and released his tadpoles in the water so his genes live on. While unfortunate for the frog, such events provide us unique insights into complex life histories of these small rain forest inhabitants.” Attribution: Andrius Pašukonis (University of Vienna).
