# Supplementary figures and images for: BMC Ecology Image Competition 2015: the winning images
Source: BMC Ecol. 2015 Jul 29;15:22. doi: 10.1186/s12898-015-0053-9 (PMC4517399; doi:10.1186/s12898-015-0053-9)

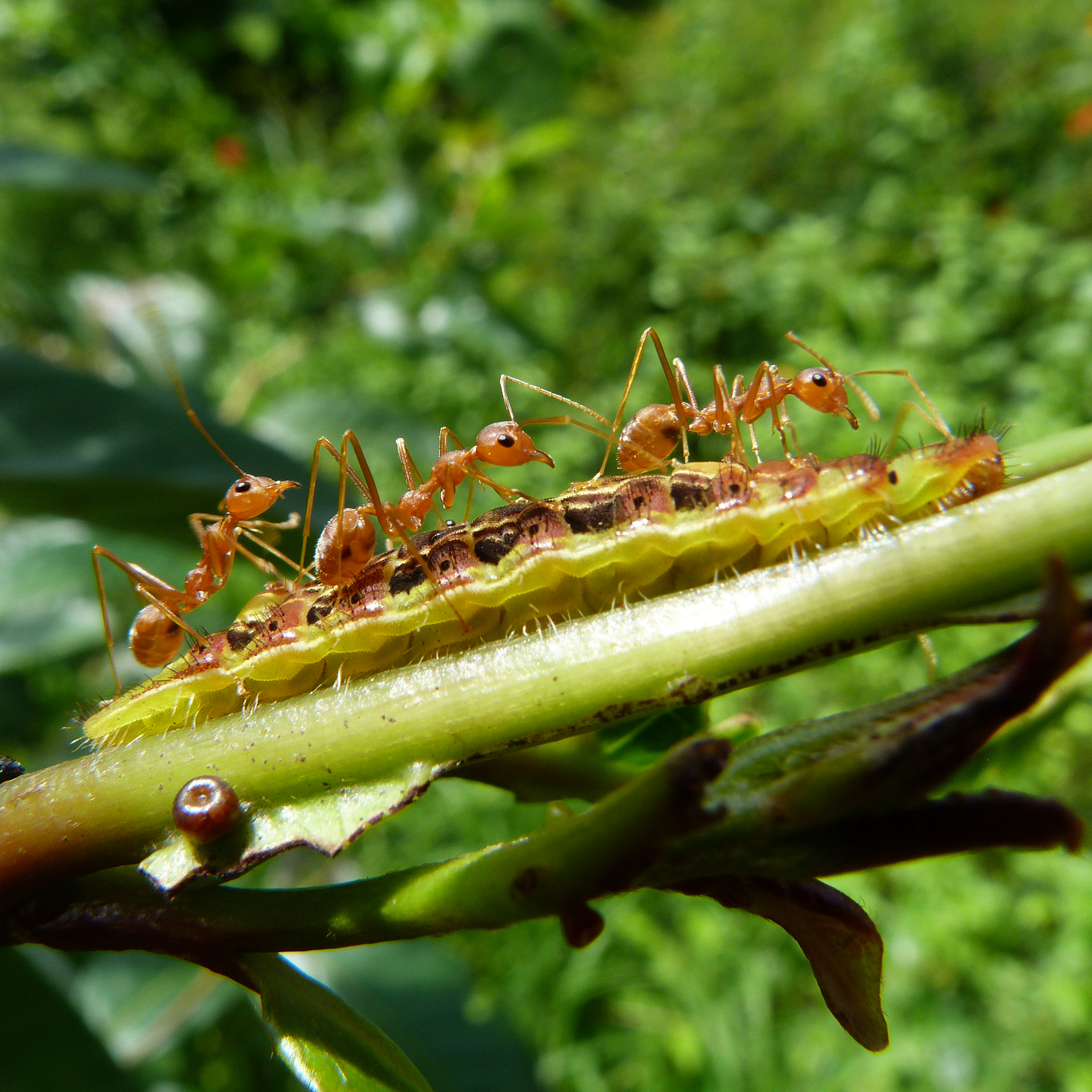

Supplement: Supplementary file 1 — “As a field researcher I keep exploring nature. One day I was in search of Weaver ants (Oecophylla smaragdina) at the University garden, I was astonished to see something interesting happening. I found a few Weaver ants encircling a caterpillar. On close inspection we found that the ants were not actually feeding on the caterpillar, but in contrast they were protecting the caterpillar. Later we got to know that this is a symbiotic relationship between the Weaver ant and the caterpillar of a butterfly family named Lycaenidae (Blues).The Caterpillars of Family Lycaenidae are known to possess glands that secrete sugary substance that attract ants. Ants swarm over the caterpillar to extract the sugary substance from it and also they are known to tickle the caterpillar with their antennas for the same purpose. In return as a reward the Weaver ants provide protection to the Caterpillar from predatory species.” Attribution: K. Vineeth Kumar (Mangalore University). [file 12898_2015_53_MOESM1_ESM.jpg]

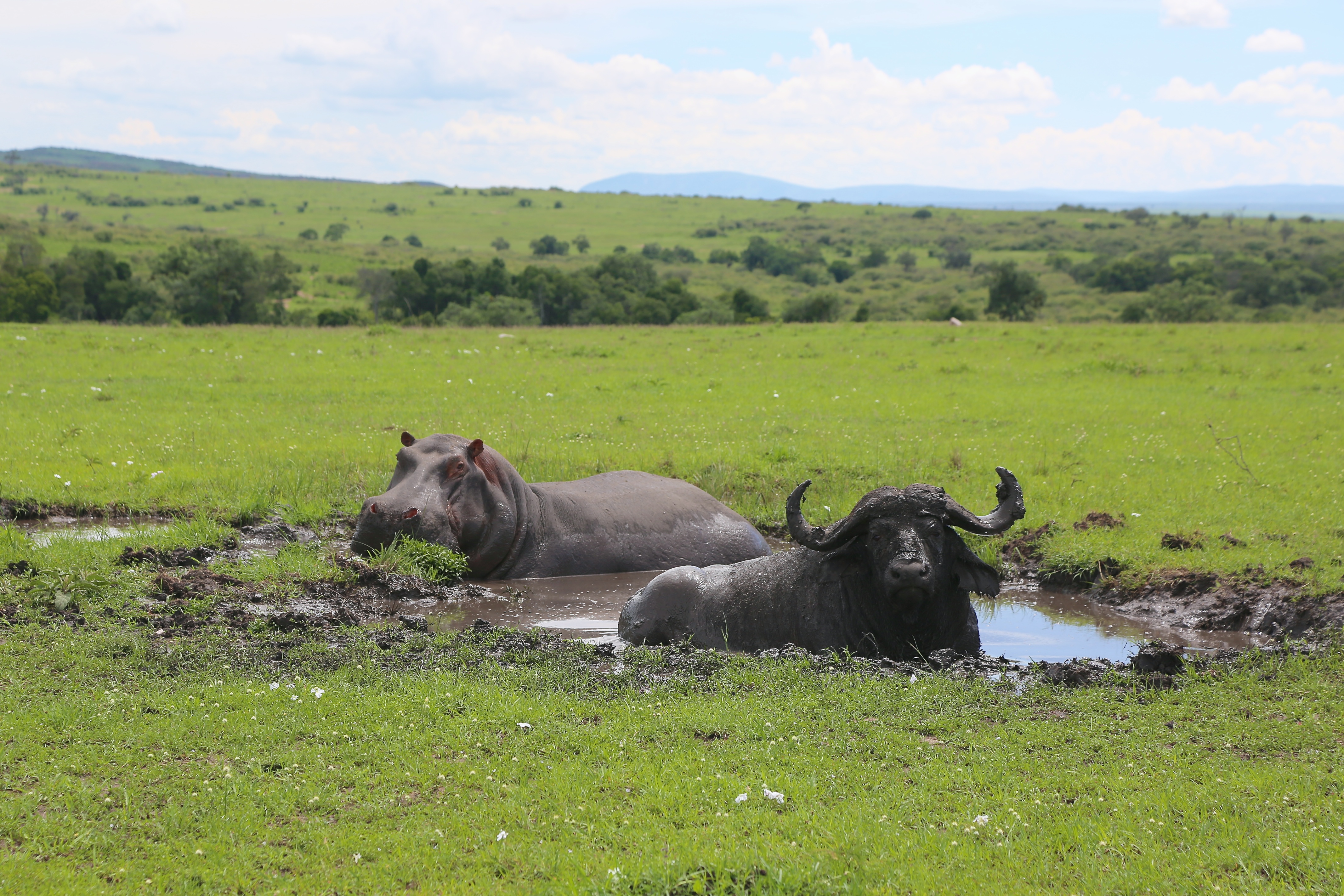

Supplement: Supplementary file 2 — “While conducting research in the Masai Mara, Kenya on the formation of mixed species groups, we spotted a hippo and buffalo sharing a rather small mud wallow. Neither species are well known for their tolerance but rather for being bad-tempered and aggressive. Nevertheless, they seemed to have come to an understanding that allowed both animals to benefit from accessing the wallow. Interestingly our research was focused on exploring which herbivore species liked to associate together and how resource availability and predation drive these aggregations (e.g. wildebeest & zebra). After more than a decade of conducting savanna fieldwork this is the first time I have seen a buffalo and hippo in such close proximity to one another.” Attribution: Graeme Shannon (University of Liverpool). [file 12898_2015_53_MOESM2_ESM.jpg]

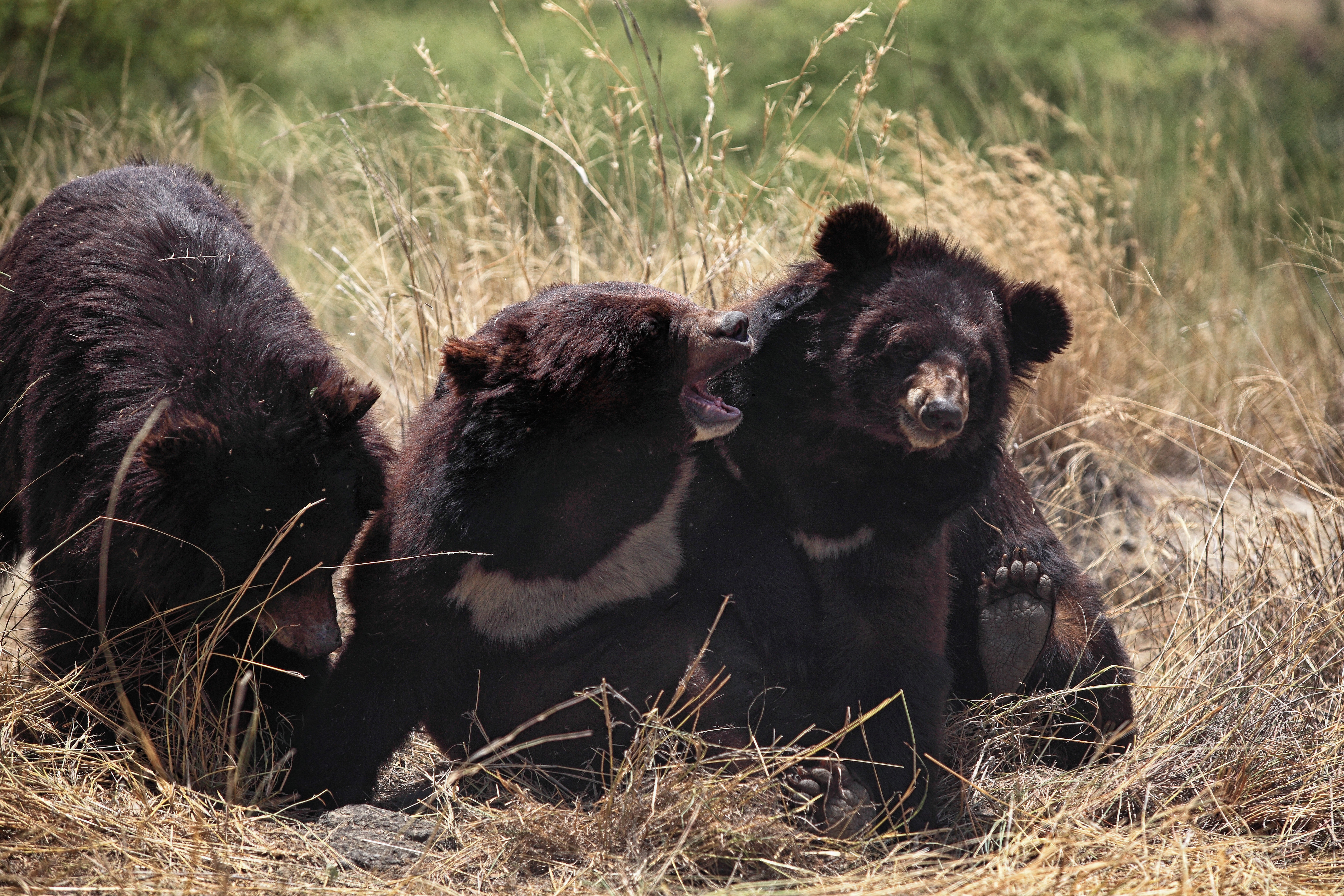

Supplement: Supplementary file 3 — “The beauty of this picture reveals the playful and socially positive interaction between 3 Asiatic black bears (Ursus thibetanus) as they enjoy the warm heat of the sun in winter season. This picture also shows their natural habitat in which they have adjusted and are enjoying it. Ursus thibetanus are found in variety of forested areas and in Pakistan they are found in Himalayan region.” Attribution: Kainaat William (Bioresource Research Centre, Pakistan). [file 12898_2015_53_MOESM3_ESM.jpg]

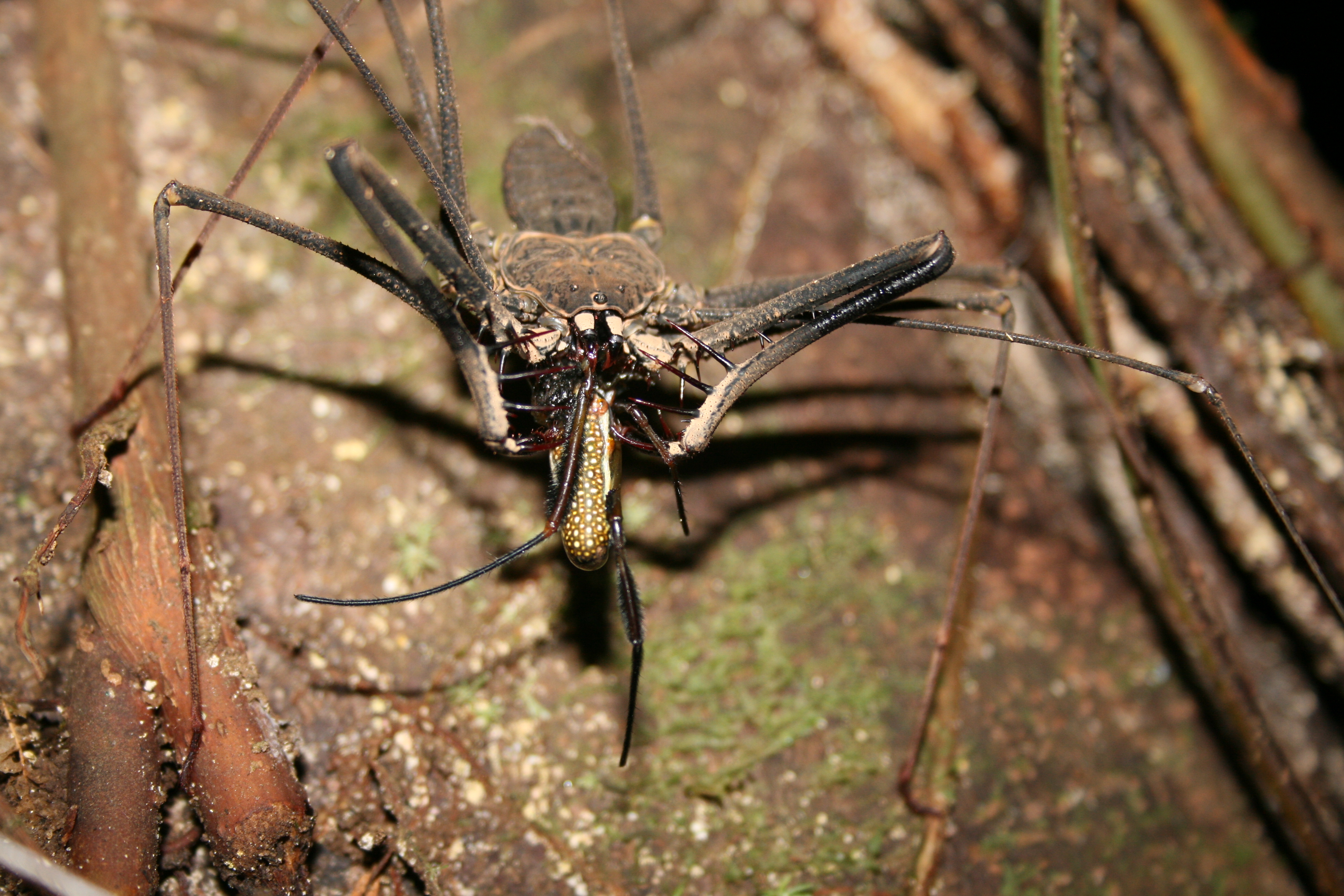

Supplement: Supplementary file 4 — “The Ecuadorian Amblypygi Heterophrynus batesii feeding on the giant golden silk orb-weaving spider Nephila sp. Amblypygi are distant relatives of spiders and scorpions outfitted with large front claws (pedipalps) and long, antenna-like front legs. The specimen pictured is larger than the human hand. While amblypygids do not produce silk, the giant orbweaver produces enormous webs with strong golden threads. This photo is the first example of amblypygi feeding on a spider, and represents intraguild predation in the community. This photo was captured at the Tiputini Biodiversity Station near Yasuni National Park in Amazonian Ecuador. Yasuni National Park is heralded as the most biodiverse place on terrestrial earth, but is threatened by pollution and development of the petroleum industry.” Attribution: Kenneth J. Chapin (University of California, Los Angeles). [file 12898_2015_53_MOESM4_ESM.jpg]

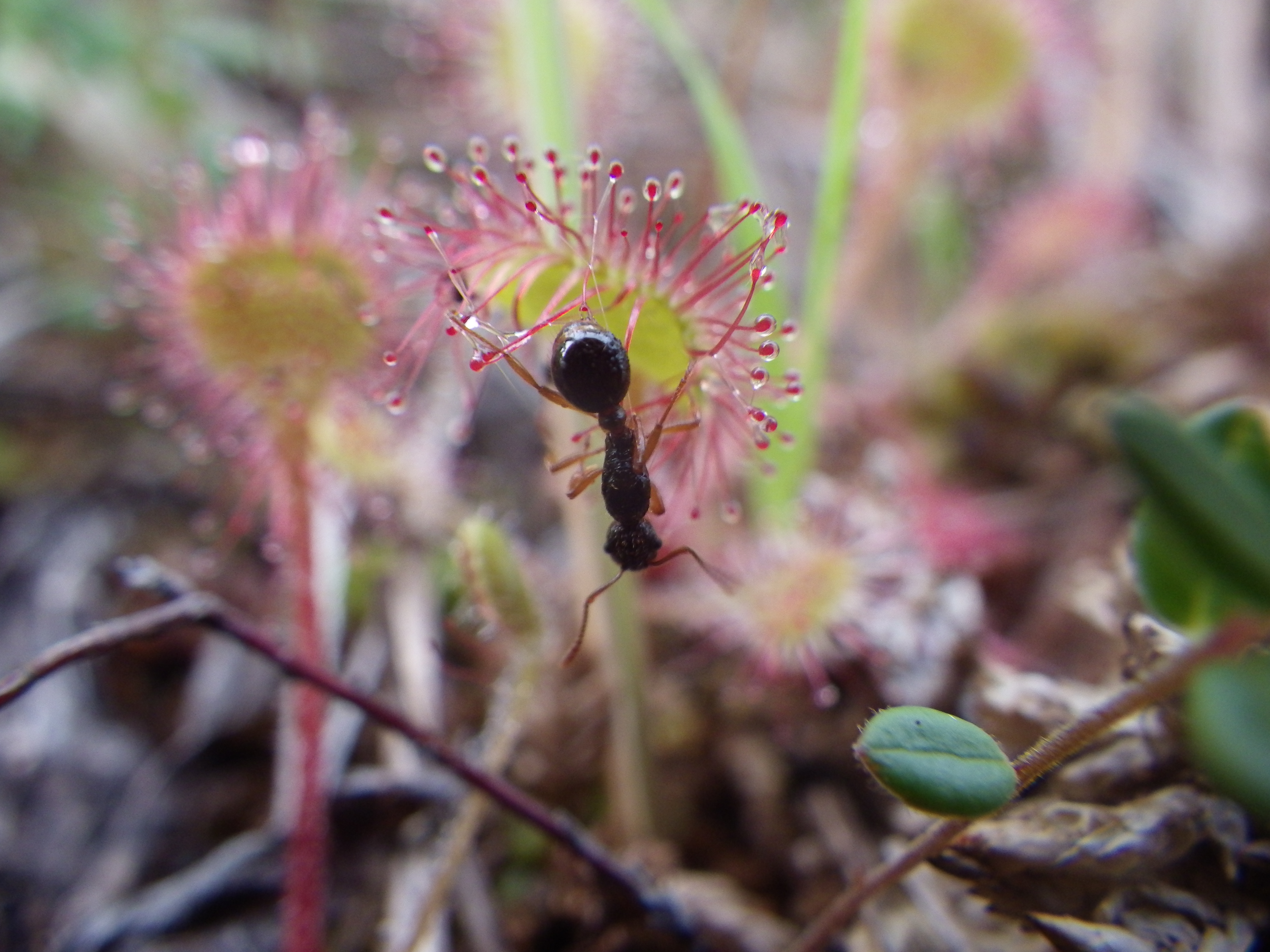

Supplement: Supplementary file 5 — “The Drosera rotundifolia is a widespread carnivorous plant in the wetlands of Japan. This picture shows an ant entangled in the sticky tentacles of the hungry plant, taken in the summer of 2013 in the Sarobetsu mire, northern Hokkaido. The more this ant moved, the more it got entangled and stuck until it became completely enveloped in the trap. The Sarobetsu mire was a coal mining site in the past, but now it is considered vulnerable area and has been converted into a conservation and research site, also attracting tourists by the colorful flowers blooming in summer.” Attribution: Harisoa Rakotonoely (Hokkaido University). [file 12898_2015_53_MOESM5_ESM.jpg]

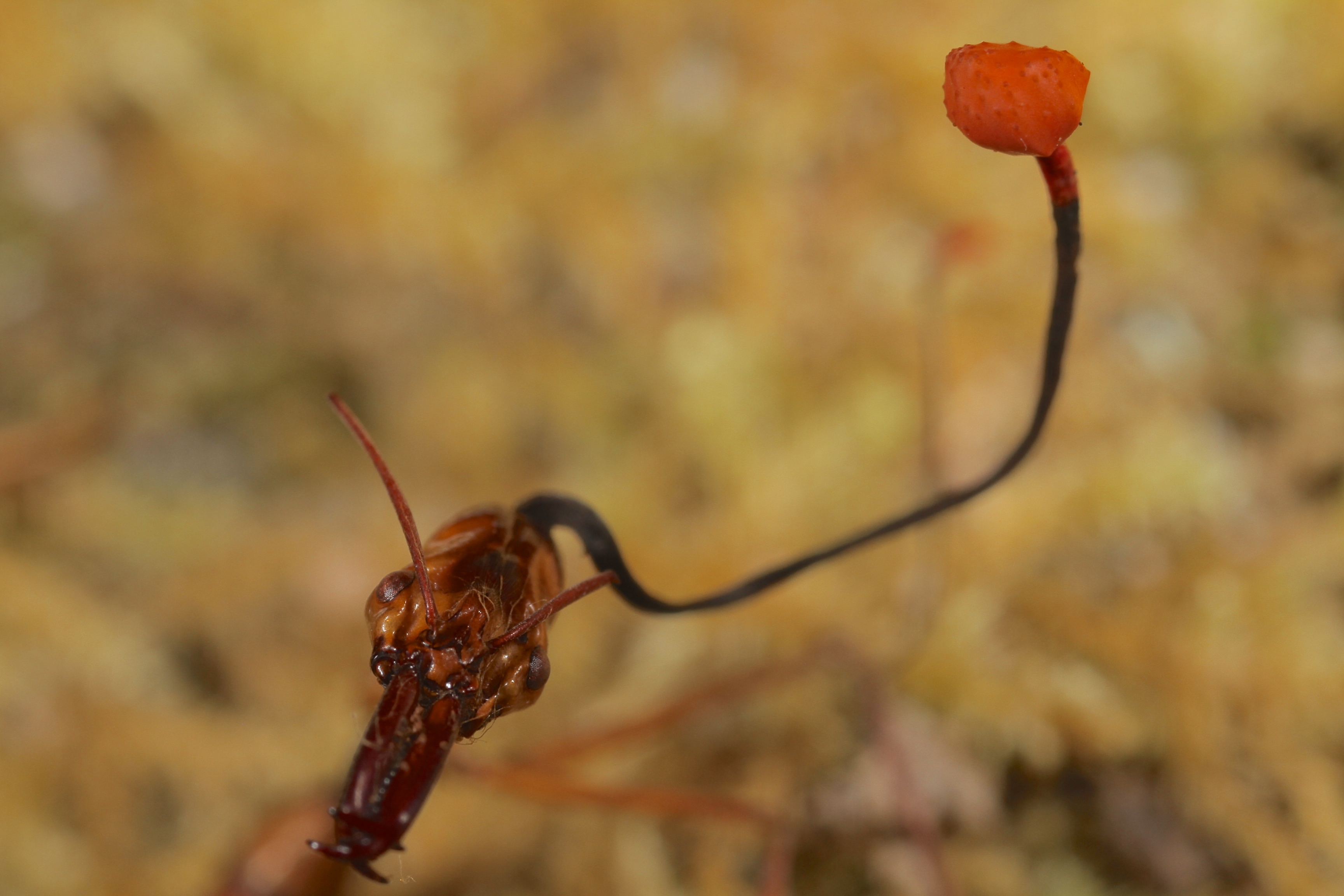

Supplement: Supplementary file 6 — “Ophiocordyceps undescribed species infecting the trap-jaw ant Odontomachus hastatus in Central Brazilian Amazon. This fungus has the ability to change ant’s behavior, making the poor insect to leave the nest to die on the mossy base of trees. There, the humidity is always constant, ensuring a permanent water supply for the parasite.” Attribution: João Araújo (Pennsylvania State University). [file 12898_2015_53_MOESM6_ESM.jpg]

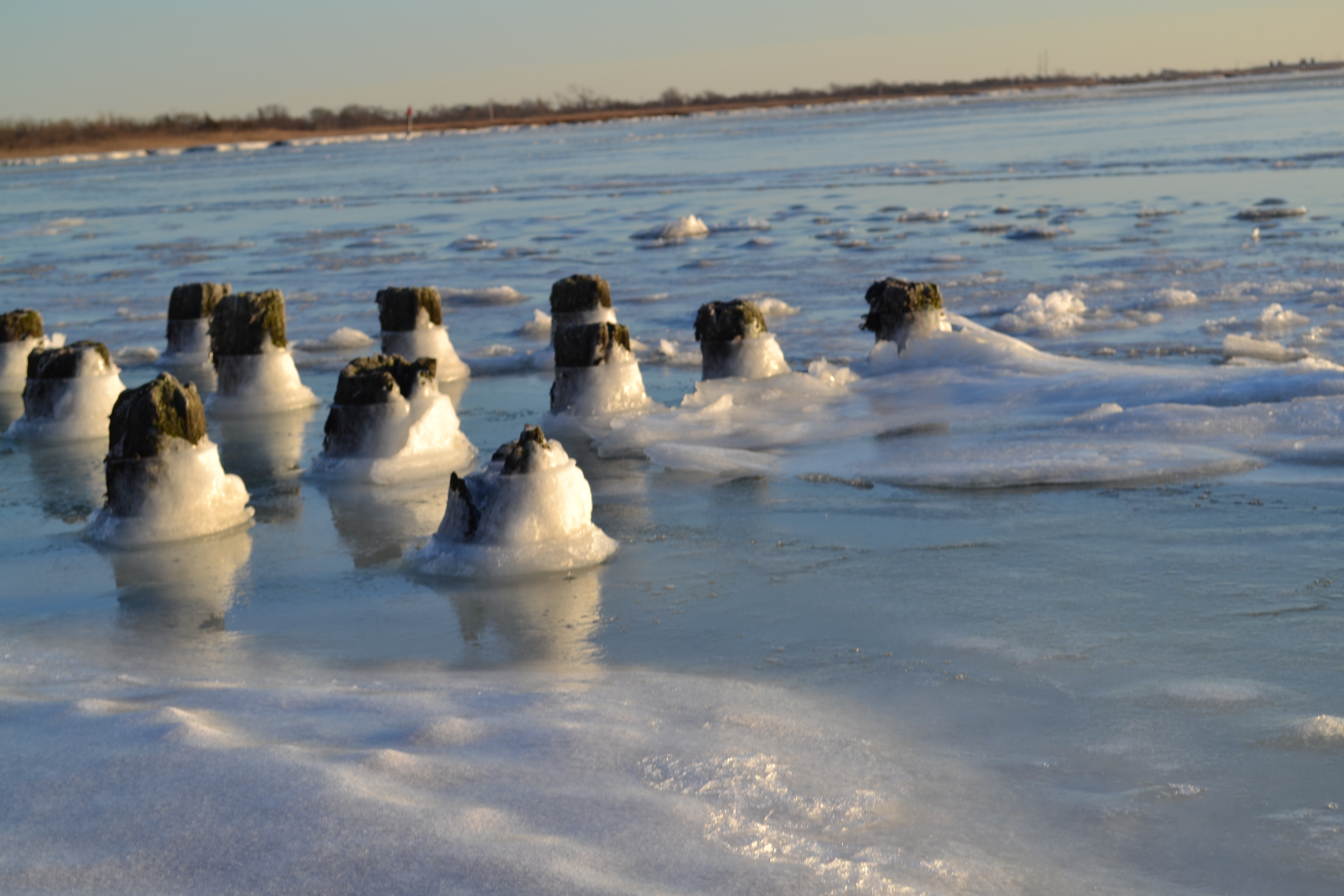

Supplement: Supplementary file 7 — “My work aims to quantify sea turtle diet on Long Island, NY. This data is obtained by analyzing stomach contents from stranded sea turtles over a 30-year time frame. Many sea turtles strand due to a condition known as cold-stunning. During the winter months, Long Islanders are encouraged to walk the shorelines as winter approaches to check for cold-stunned sea turtles. This photo, captured at Wantagh Park on Long Island, NY, depicts how the ecosystem that is home to sea turtles in the summer months is altered with the change of seasons. This landscape is a potential stranding site for sea turtles that do not migrate down south before the change of season. As Long Island waters cool, sea turtles begin their migration. Those that do not make this migration in time are subjected to freezing conditions, depicted above, and become cold-stunned. If the sea turtle does not survive this condition, we quantify the stomach contents to analyze sea turtle diet in Long Island waters. This research not only provides insight into sea turtle diet, but also paints a picture of the ecosystem these species are present in. We can analyze which prey species are available in the surrounding ecosystem, as well as their population trends over time.” Attribution: Christina Giordano (Stony Brook University). [file 12898_2015_53_MOESM7_ESM.jpg]

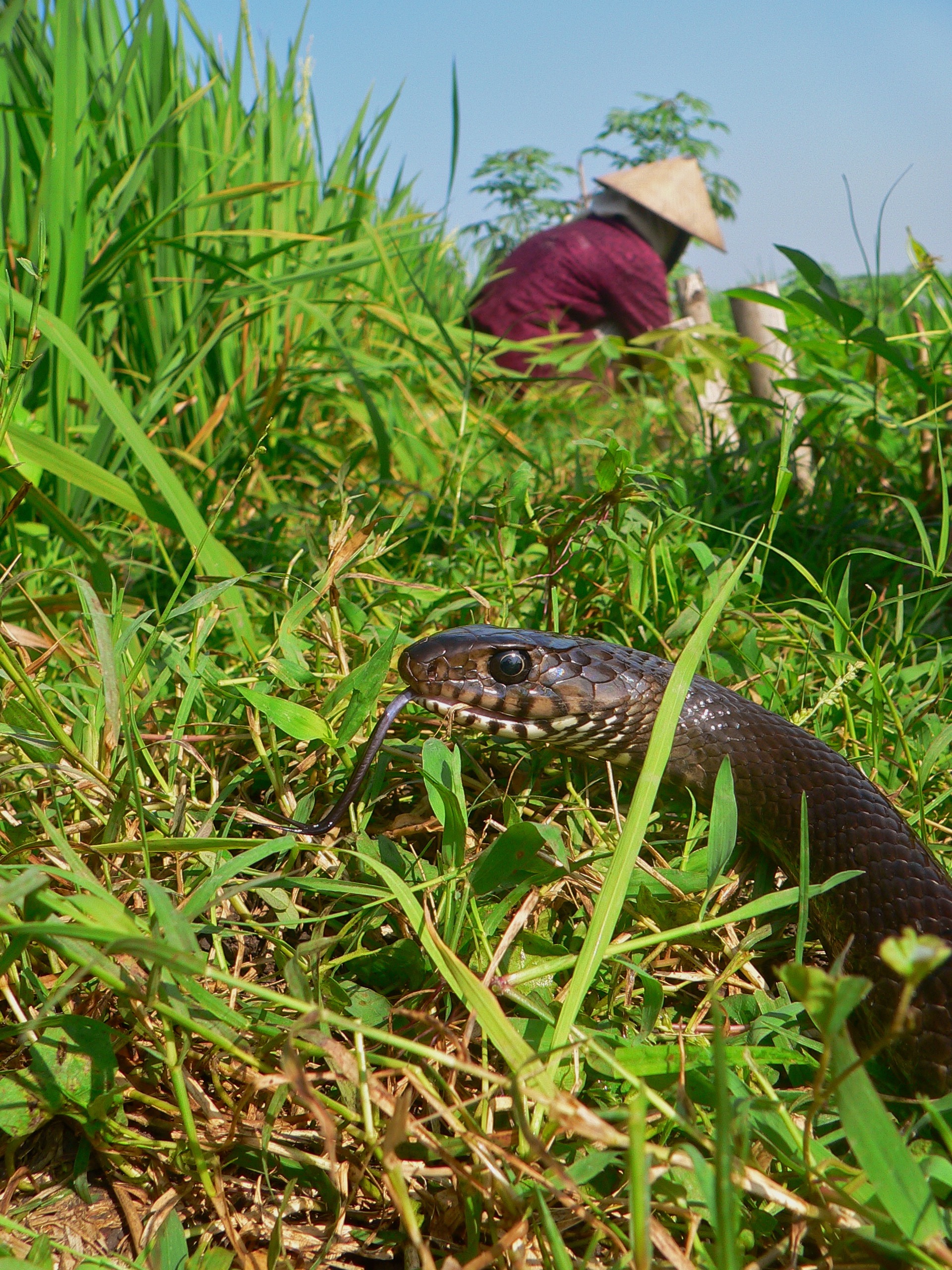

Supplement: Supplementary file 8 — “The Oriental Rat Snake (Ptyas mucosa) is distributed from central Asia to East and South East Asia. The diurnal active species mainly preys on frogs and rodents in open agro-ecosystems. The photo shows an adult specimen in search for food close to a paddy field worker in Central Java (Indonesia). Since the early 20th century, the species is involved in the international skin industry. In 1990 the species was listed on Appendix II of CITES (Convention on International Trade in Endangered Species of Fauna and Flora) that regulates trade by an export permit, which is granted through the relevant management authorities. Particularly populations on Java (Indonesia) are intensively harvested for their skins. The 2015 export quota from Indonesia is 89 559 skins and skin products plus 441 live specimens.” Attribution: Mark Auliya (Helmholtz Centre for Environmental Research GmbH-UFZ). [file 12898_2015_53_MOESM8_ESM.jpg]

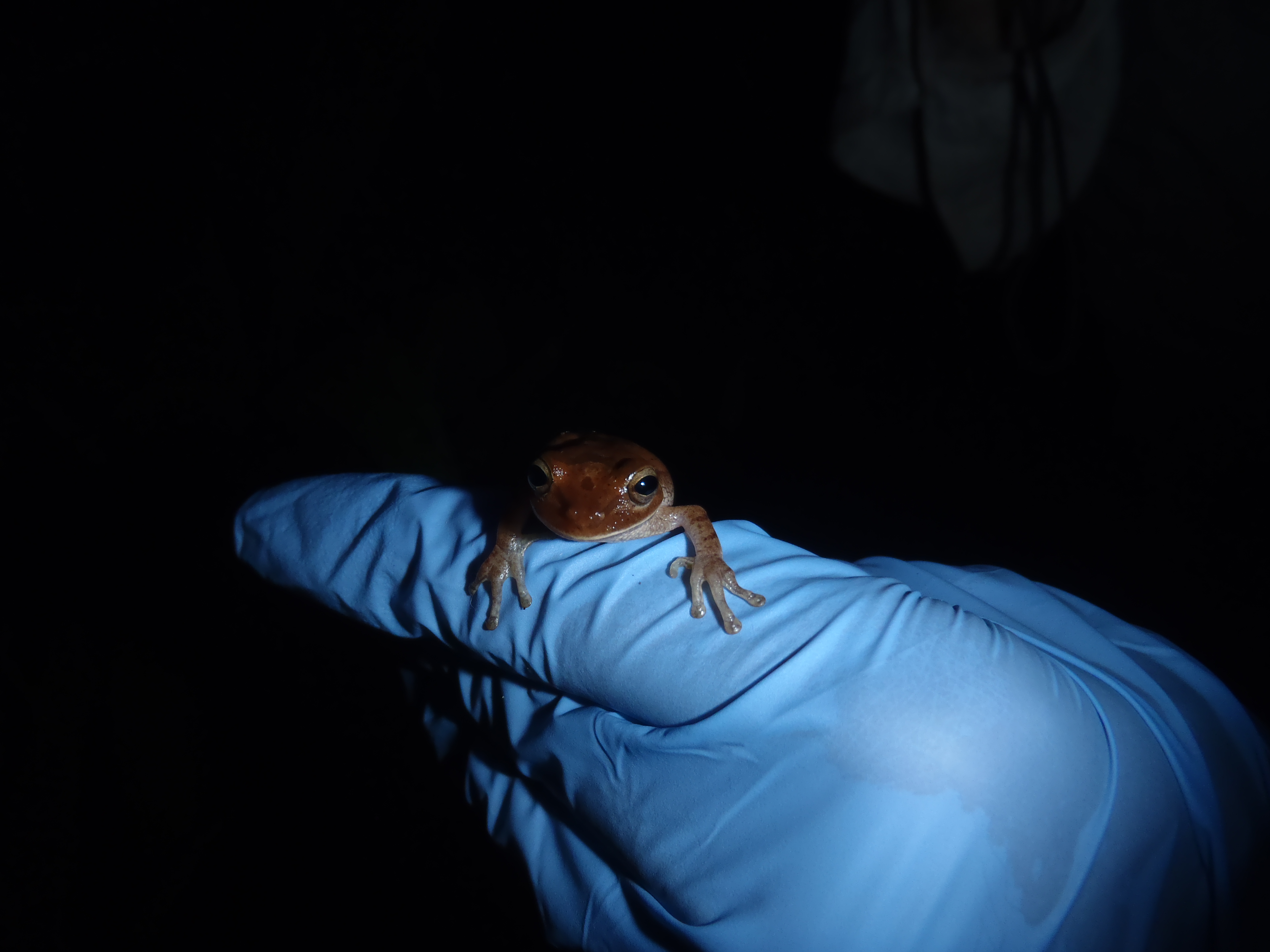

Supplement: Supplementary file 9 — “More than one-third of amphibians are threatened with extinction globally. In certain parts of the world, such as Central America, this number may reach as high as one-half. A major contributor to amphibian decline in these areas is a devastating disease known as chytridiomycosis. Chytridiomycosis is a fungal infection that is believed to cause death by disrupting an amphibian’s ability to regulate water, oxygen, and electrolytes through its skin. The disease is highly infectious and human activity has greatly increased the spread of the fungus, which now occurs worldwide. Although certain species exhibit relative immunity, the disease has been implicated in the catastrophic decline or complete extinction of no less than 200 species. In places hit hardest by chytridiomycosis the disease may eliminate over half of all species from an area. Remaining species experience declines of up to 80%. These crashes unfold in as little as six months, with recovery time estimated at approximately 15 years. Unique to amphibians, chytridiomycosis is the only disease known to cause such massive decline in species not otherwise at risk of extinction. Additionally, declines caused by chytridiomycosis are believed to represent the largest loss of biodiversity attributable to disease. Fortunately, the spread of the chytrid fungus can be predicted once it has struck, its presence can be detected with diligent monitoring, and amphibian populations can be brought into captivity in order to avoid the disease. Such efforts, however, are massive undertakings which require widespread collaborations among extremely dedicated conservationists in multiple fields of study. Among those who work with amphibians first-hand extreme care must be taken not only to prevent harming delicate individuals, but also to prevent any possibility of spreading the disease among individuals or across habitats.” Attribution: Mark Spangler (University of Alaska). [file 12898_2015_53_MOESM9_ESM.jpg]

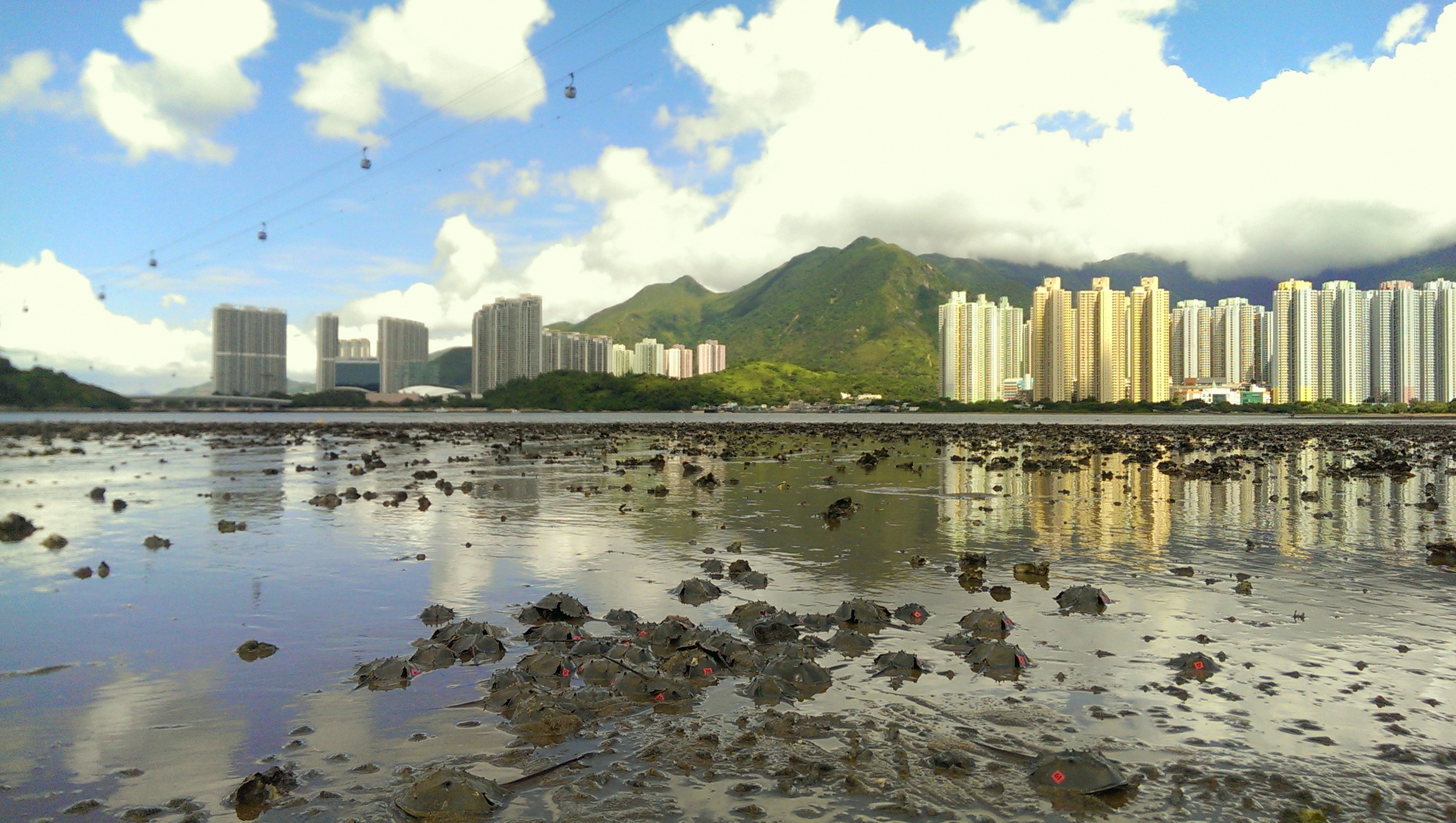

Supplement: Supplementary file 10 — “My research is focused on the ecology and conservation of an ancient marine arthropod, horseshoe crab Tachypleus tridentatus in which the oldest fossil could probably be dated back to Late Ordovician Period. Their populations were in dramatic declines in many Asian places due to habitat destructions for coastal developments and harvest pressure for biomedical applications and consumption. Because of the lack of scientific data, this species is listed as Data Deficient on the IUCN Red List of Threatened Species, thus the animal is not being protected by neither the international treaties nor the local law. The picture was taken during a research on the home range area utilized by the juveniles on an intertidal mudflat in Hong Kong, in which the shore is opposite to largely reclaimed Hong Kong International Airport and the newly developed Tung Chung Town, separated by a narrow waterway. On top of the shore is the Ngong Ping Cable Car connecting Tung Chung Town and the famous tourist attractions, Po Lin Monastery and the Tian Tan Buddha. During the study in summer 2014, the juveniles were labeled by a waterproof red-colored plastic tag and allowed to move for foraging and normal daily activities. Their recaptured locations were recorded for home range calculations. The aim of this study was to provide preliminary data on the movement patterns and space utilization of juvenile horseshoe crabs in the field, which may be useful for the possible designation of conservation area in the future. While there is hardly any proposal for the habitat protection for this animal group, Tung Chung New Town Extension Project has been proposed and the EIA study was on the public inspection stage when the home range study was still conducting. Who will win in this space-competing game? Should be the Humans, with no doubt.” Attribution: Billy Kit Yue Kwan (City University of Hong Kong). [file 12898_2015_53_MOESM10_ESM.jpg]

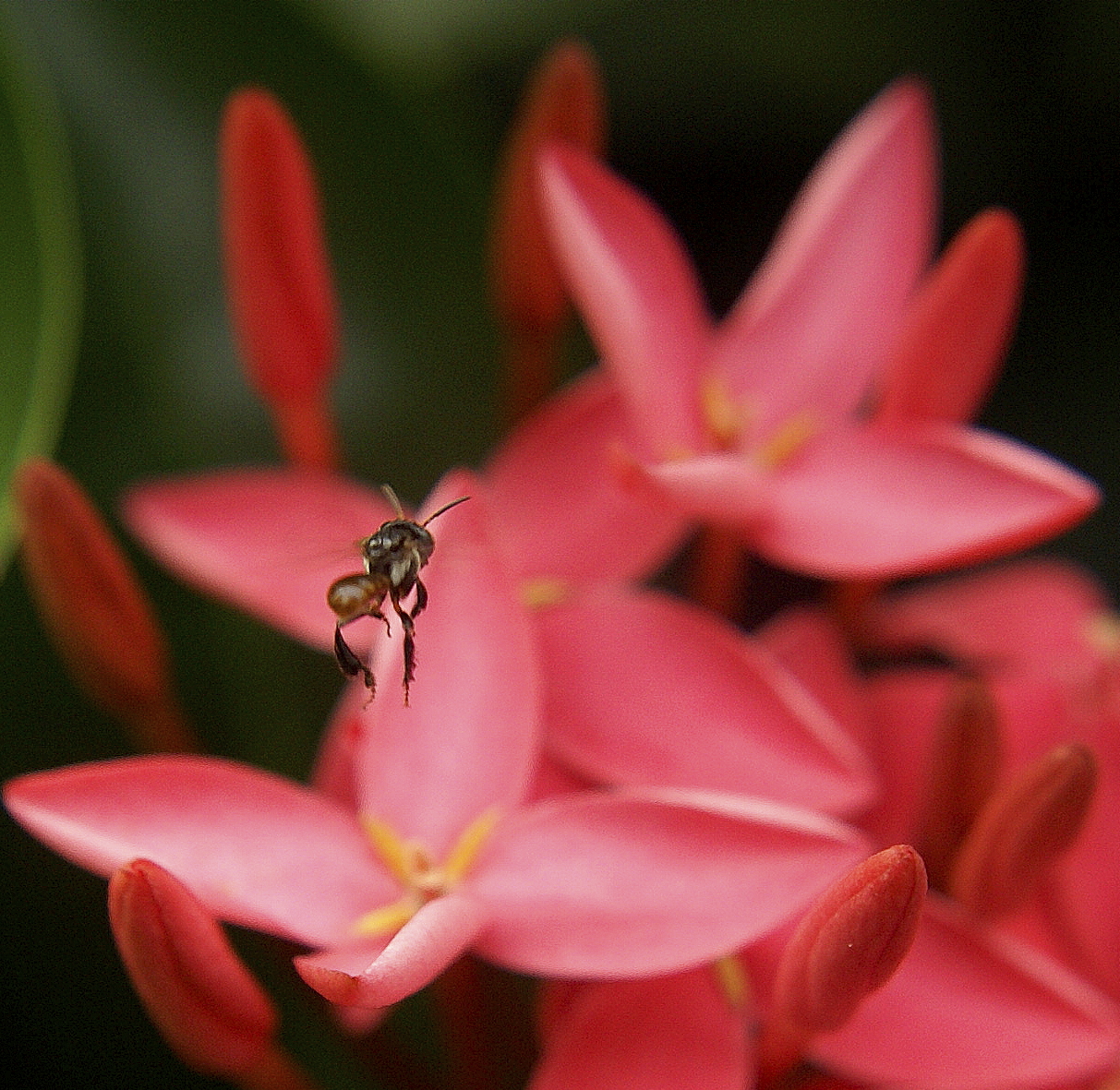

Supplement: Supplementary file 11 — “Conservation of pollinators consists of bees and native bees in Malaysia. With the number of bees in decline because of pesticide and colony collapse disorder (ccd), the need for conservation is getting more and more important. Compared to the bees, stingless bees are another pollinator in Malaysia which has currently gained popularity in Malaysia. The stingless bees Trigona sp. is being utilized as pollinator and also for its honey.” Attribution: Mohd Masri bin Saranum (Malaysian Agricultural Research and Development Institute). [file 12898_2015_53_MOESM11_ESM.jpg]

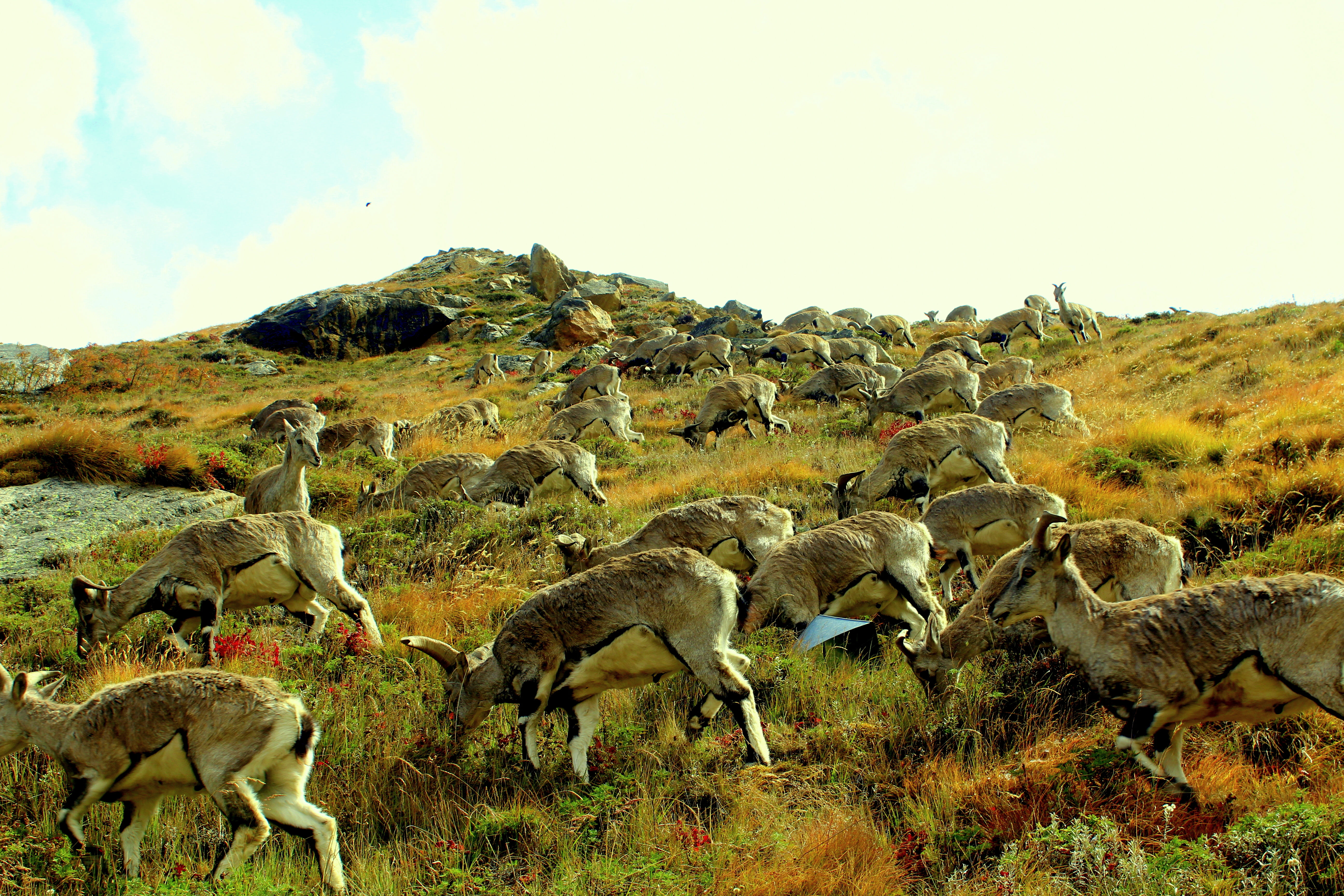

Supplement: Supplementary file 12 — “Pseudois nayaur (Himalayan Blue Sheep) is a goat antelope found in greater Himalayas. This photograph is taken in Valley surrounding the Gangetic glacier at a height of 5,600 meters above the sea level. They are preferred meal for the snow leopard (Uncia uncia).They are exceptional in climbing high mountains and show excellent camouflage capability with the surrounding environment. P. nayur remain in large groups and shows aggressive territorial behavior and the species play a crucial role in ecology of the surrounding nutrient poor habitat.” Attribution: Vineet Kumar Singh (University of Delhi). [file 12898_2015_53_MOESM12_ESM.jpg]

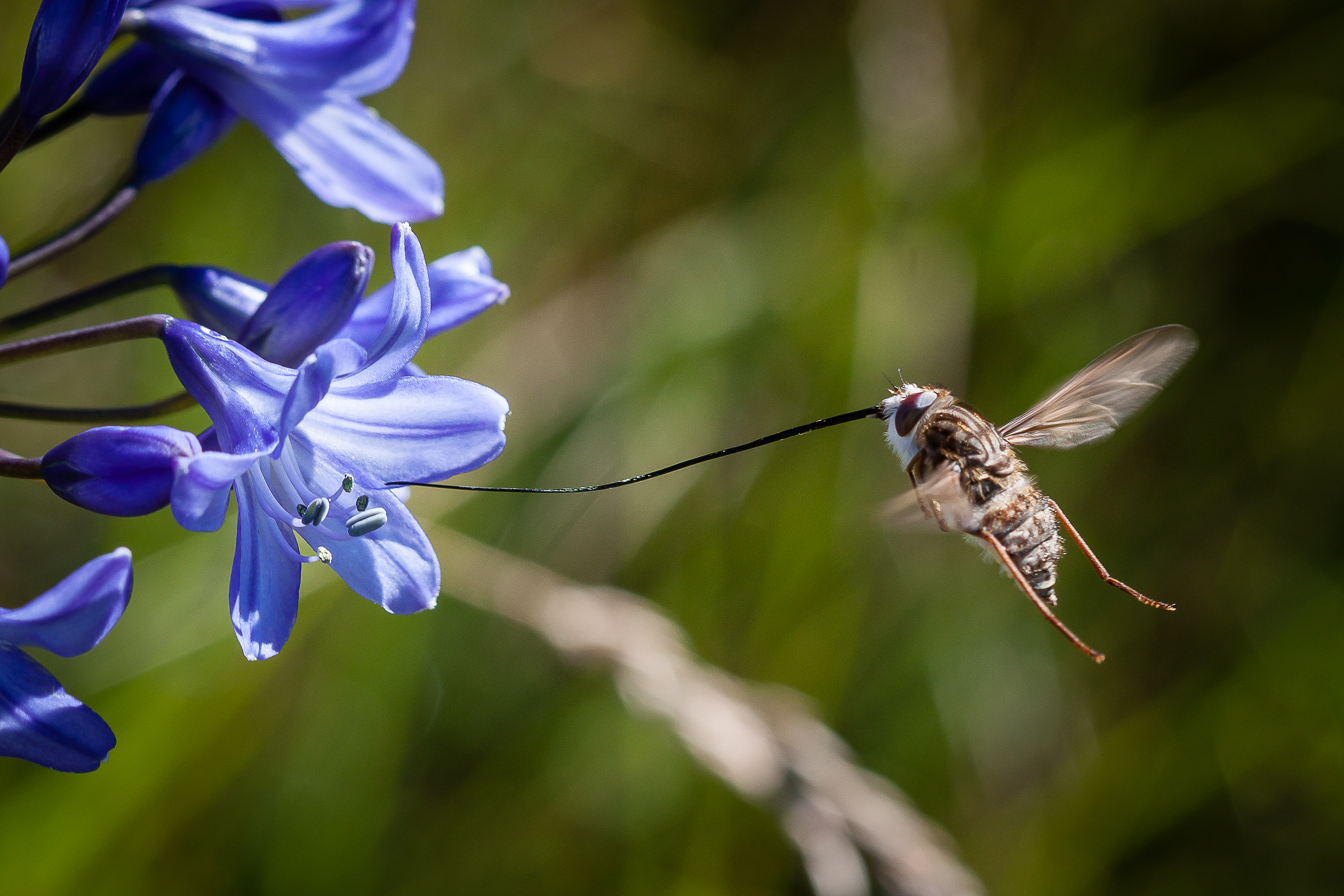

Supplement: Supplementary file 13 — “The remarkable proboscis of South Africa’s long-tongue fly (Prosoeca ganglbaueri) can attain lengths of over 50 mm. Due to the co-evolutionary feedback that drives length in both fly tongues and floral nectar tubes, this specialist nectar-feeder is now the exclusive pollinator for over 20 species of long-tubed flower. This however does not stop it from nectar robbing of shorter-tubed species such as the Agapanthus seen here. Given the remarkable diversity of plants that have come to rely on them as sole pollinator, the long-tongue flies of Southern Africa are truly an exemplar keystone species.” Attribution: Michael Whitehead (Australian National University). [file 12898_2015_53_MOESM13_ESM.jpg]

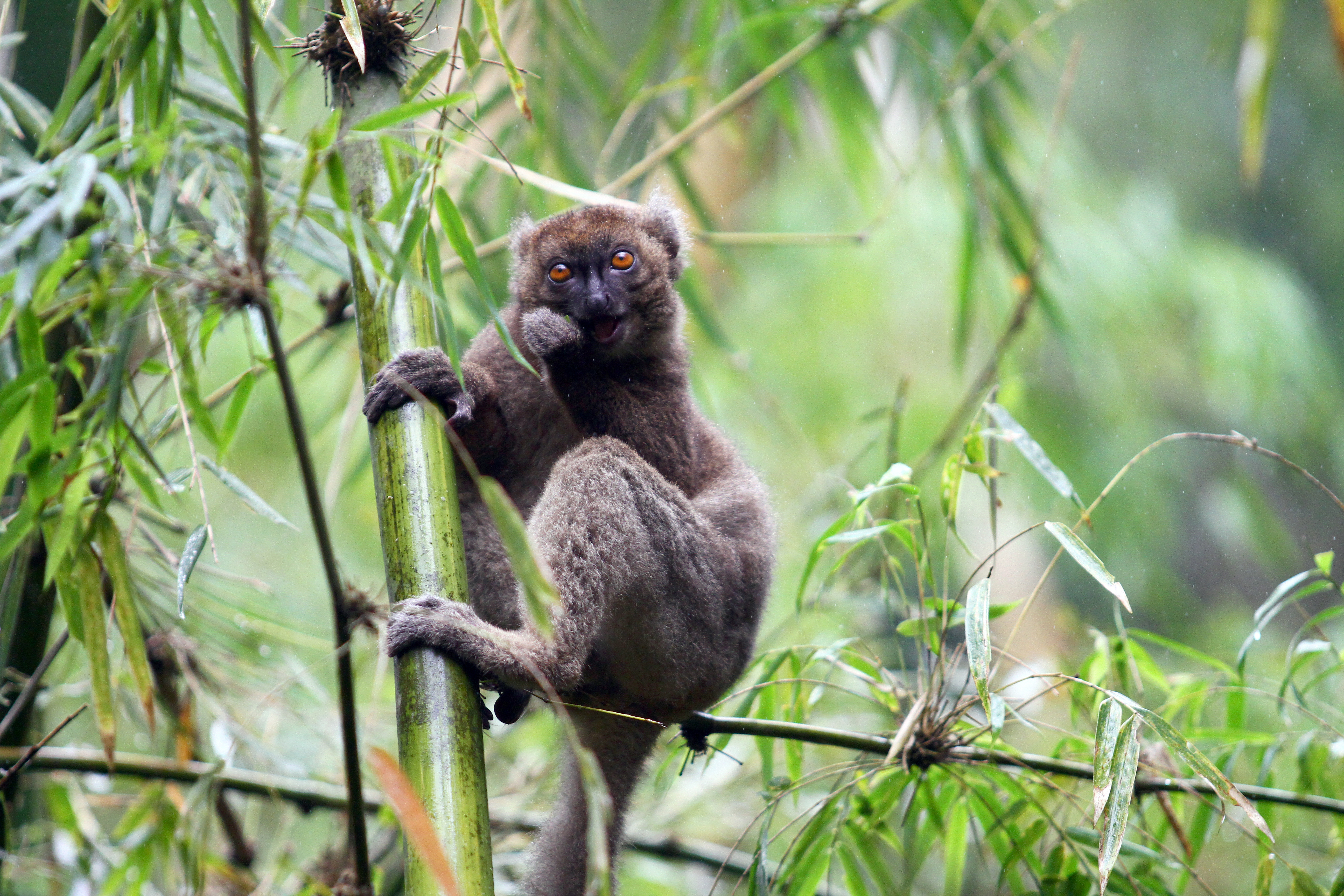

Supplement: Supplementary file 14 — “A greater bamboo lemur (Prolemur simus) feeds on bamboo leaves during a rainstorm in Kianjavato, Madagascar. Madagascar giant bamboo (Cathariostachys madagascariensis), the primary food source of this species, contains cyanogenic glycosides that are highly toxic to most mammals. On average, this lemur daily consumes ten times the amount of cyanide that would be lethal to other mammals of similar size. While it is not yet known how this primate is able to safely ingest the toxins, its physiological adaptations have allowed for the utilization of a widely available resource with little competition from other species. Greater bamboo lemurs are critically endangered and occupy only 1-4% of their original home range in southeastern Madagascar. Leading threats include habitat loss and fragmentation as a result of slash-and-burn agriculture, mining, and illegal logging.” Attribution: Peggy Boone (University of Wisconsin-Madison). [file 12898_2015_53_MOESM14_ESM.jpg]

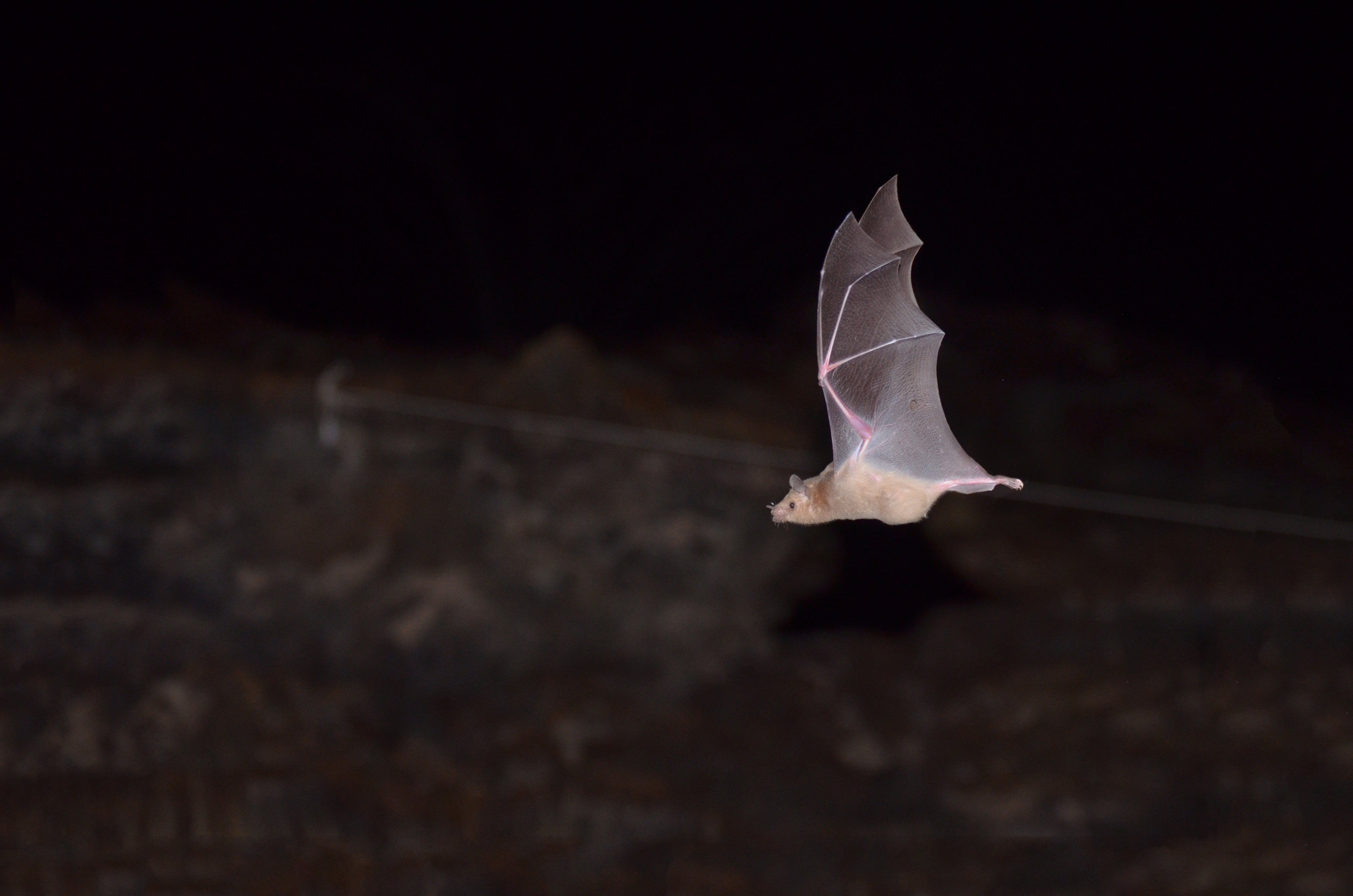

Supplement: Supplementary file 15 — “This is a pregnant Leptonycteris yerbabuenae. This female carried her baby in her womb from the coast of Jalisco to the Sonoran desert, where, it met another 100,000 to 300,000 females to give birth in a maternity cave in the Biosphere Reserve of Pinacate and Great Altar Desert in Sonora, Mexico. This bat was in the endangered species list in Mexico (NOM-059), and after almost 20 years of conservation efforts, scientific research and education to the communities, last year it left the list because its populations are stabilized or increased. Nowadays the people in the Laboratory for Terrestrial Vertebrates Conservation in the Ecology Institute (UNAM) are still monitoring this and other colonies to keep an eye on the species wellness.” Attribution: Alma Rosa Moreno Pérez (Ciudad Universitaria). [file 12898_2015_53_MOESM15_ESM.jpg]

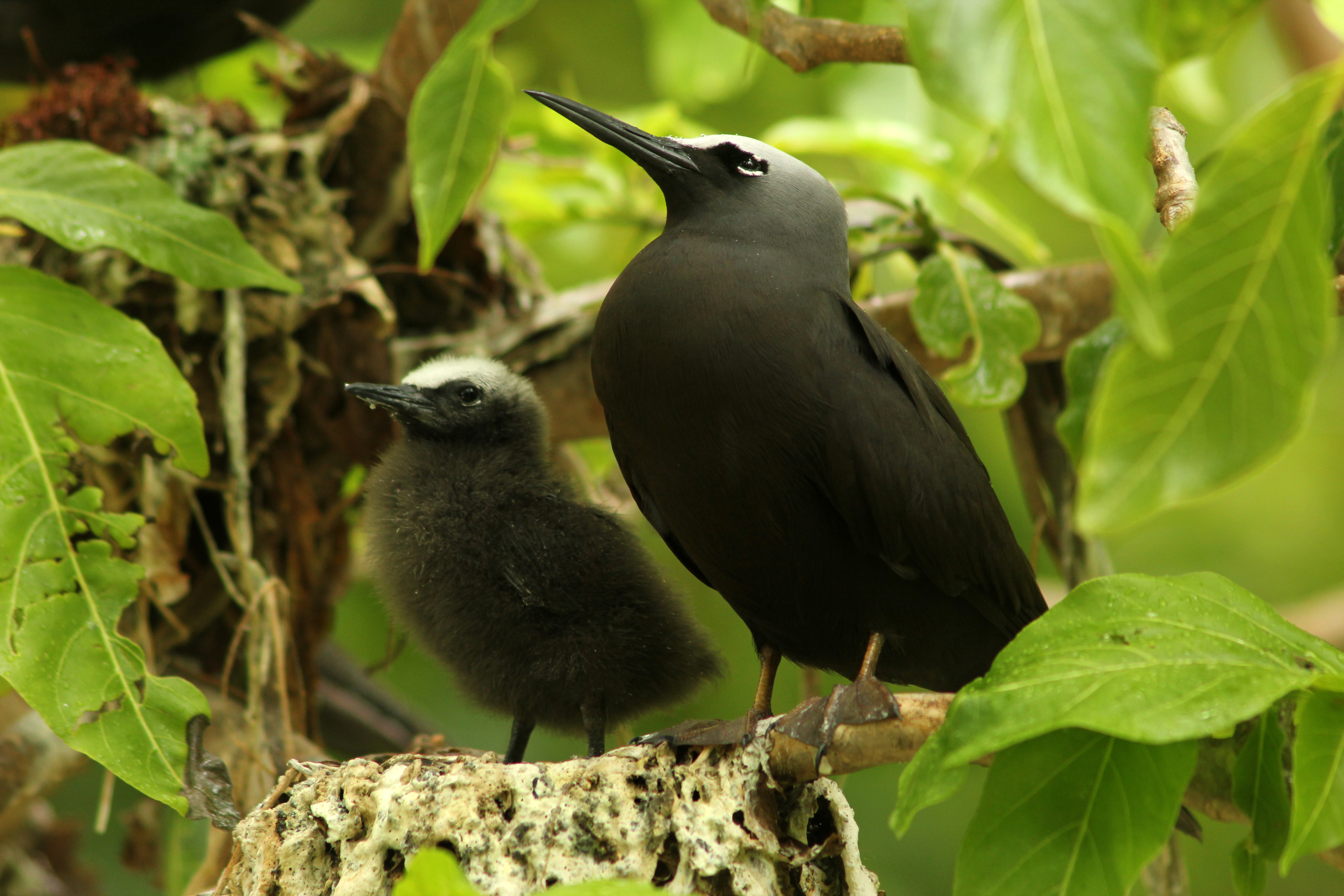

Supplement: Supplementary file 16 — “Heron Island, Great Barrier Reef-February 2015. A proud noddy tern chick and parent stand guard at their Pisonia tree nest on Heron Island, Great Barrier Reef, Australia. Known for their nodding behavior during their courtship dances, the black tern Anous minutus normally lays one egg each breeding season. But this chick is a latecomer from a second round of offspring after a heavy storm brought down many of the original nests made in the peak breeding season. Strong winds are not the only threat to these birds; other dangers lurk closer to home. Pisonia grandis forms a lush forest on the small coral cay, partly supported by the guano of the large bird population that it fosters by providing shelter and nest building material. Yet its sticky fruits are a deadly trap for the unwary youngsters or even the adult noddies. Death by immobilization is so common on the forest floor that it is hypothesized as an evolutionary strategy of the tree for fertilizing the soil with decaying animal matter. Is the onshore productivity of the forest linked to the productivity in the surrounding waters, which host a colorful forest of their own? The coral reef encircling the lagoon is what brings marine biologists like myself to the island for scientific research. But even if you spend most of your time underwater at Heron Island you are guaranteed to encounter the noddies, as there are about 80,000 of them on the small coral cay at any time. You would think the island should be renamed after them, and they would nod in approval.” Attribution: Michelle Achlatis (University of Queensland). [file 12898_2015_53_MOESM16_ESM.jpg]

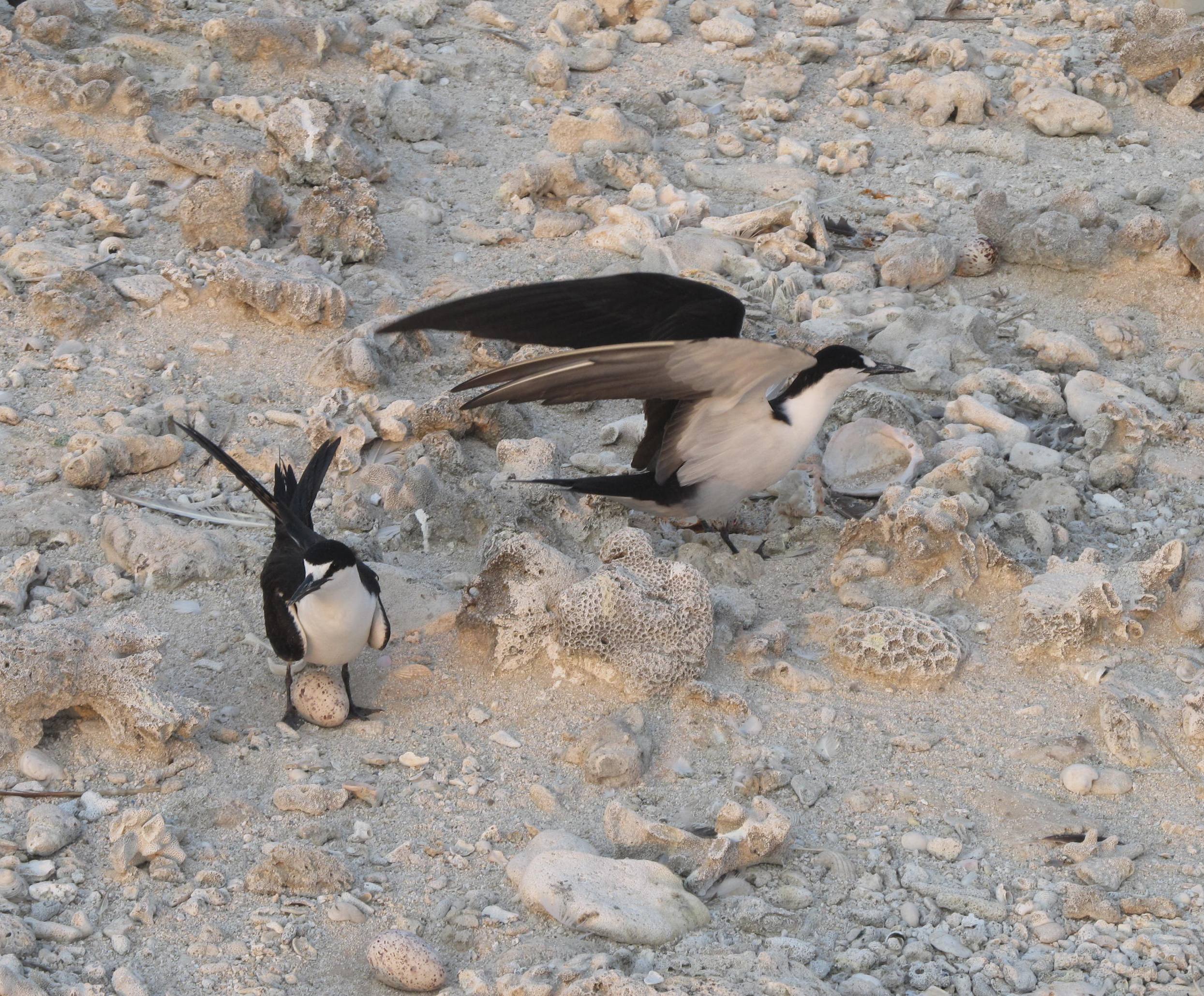

Supplement: Supplementary file 17 — “A pair of Sooty Terns incubating in the only known extant colony of seabirds in Pitti Island, Lakshadweep, India. Here the pair was seen taking turns to incubate and shade the only egg. The egg would be incubated for approximately for a period of 29 days. During this period the bird is attentive over 95% of the time incubating when it is cool and shading when it is hot.” Attribution: Ravichandra Mondreti (Centre for Ecology and Functional Evolution, CNRS). [file 12898_2015_53_MOESM17_ESM.jpg]

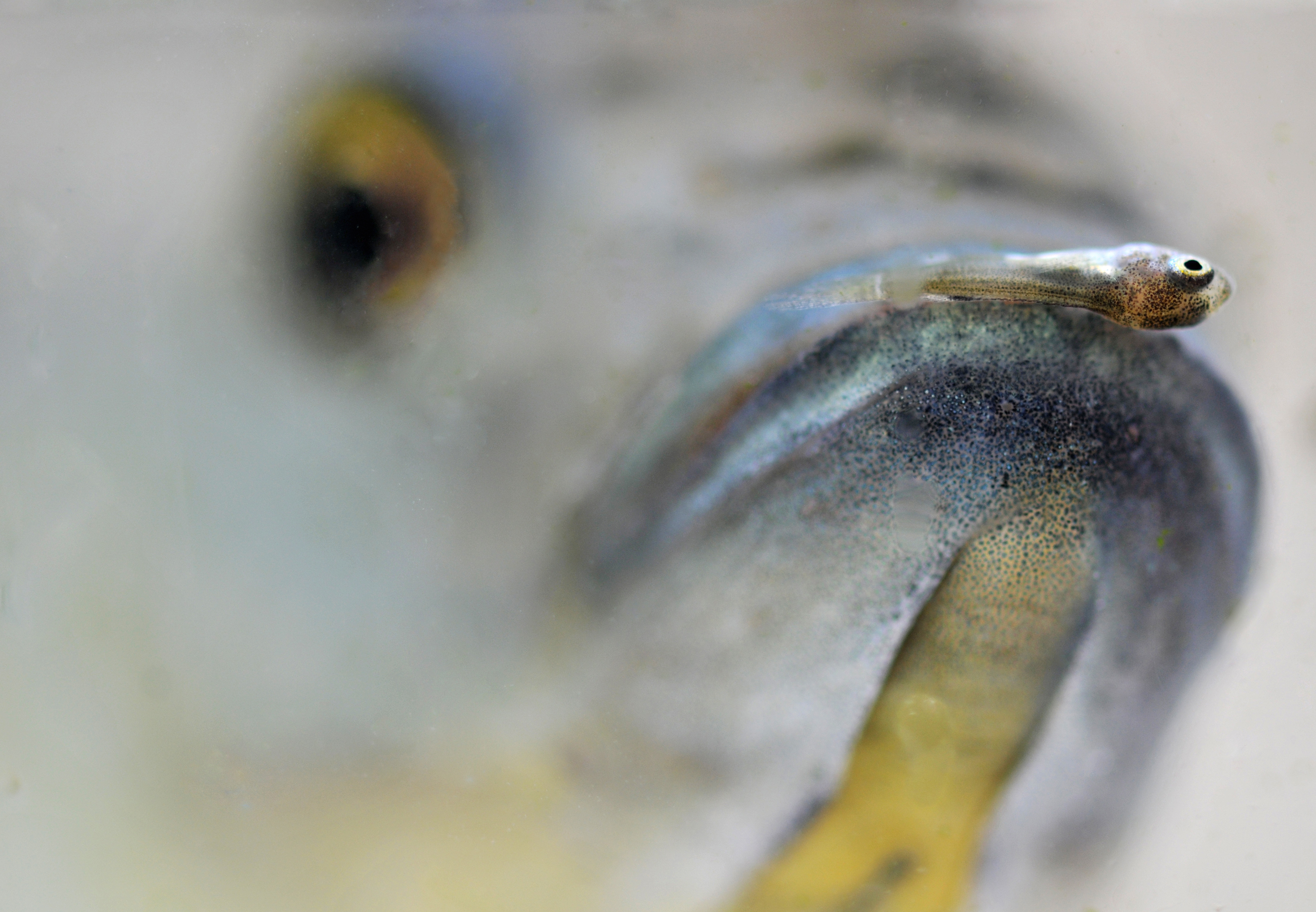

Supplement: Supplementary file 18 — “Tilapia are widely used in aquaculture, and for research. The euryhaline Mozambique tilapia (Oreochromis mossambicus) has a remarkable capacity to adapt to environments ranging from fresh water to double-strength seawater. For this reason, the Mozambique tilapia is a good model to study osmoregulation and the effects of environmental salinity on growth and reproduction. The Mozambique tilapia is a mouth-brooding species, originally found in the estuarine areas of southeast Africa. The female incubates the fertilized eggs in its mouth. During this incubation period, the fertilized eggs develop into yolk sac fry, which after 5 to 8 days become free swimming, as the swim bladder develops. Following absorption of the yolk, they become juveniles and no longer seek shelter in the female’s mouth. The image depicts a brooding female Mozambique tilapia with a yolk sac fry resting on its lower lip.” Attribution: Andre P. Seale (University of Hawaii). [file 12898_2015_53_MOESM18_ESM.jpg]

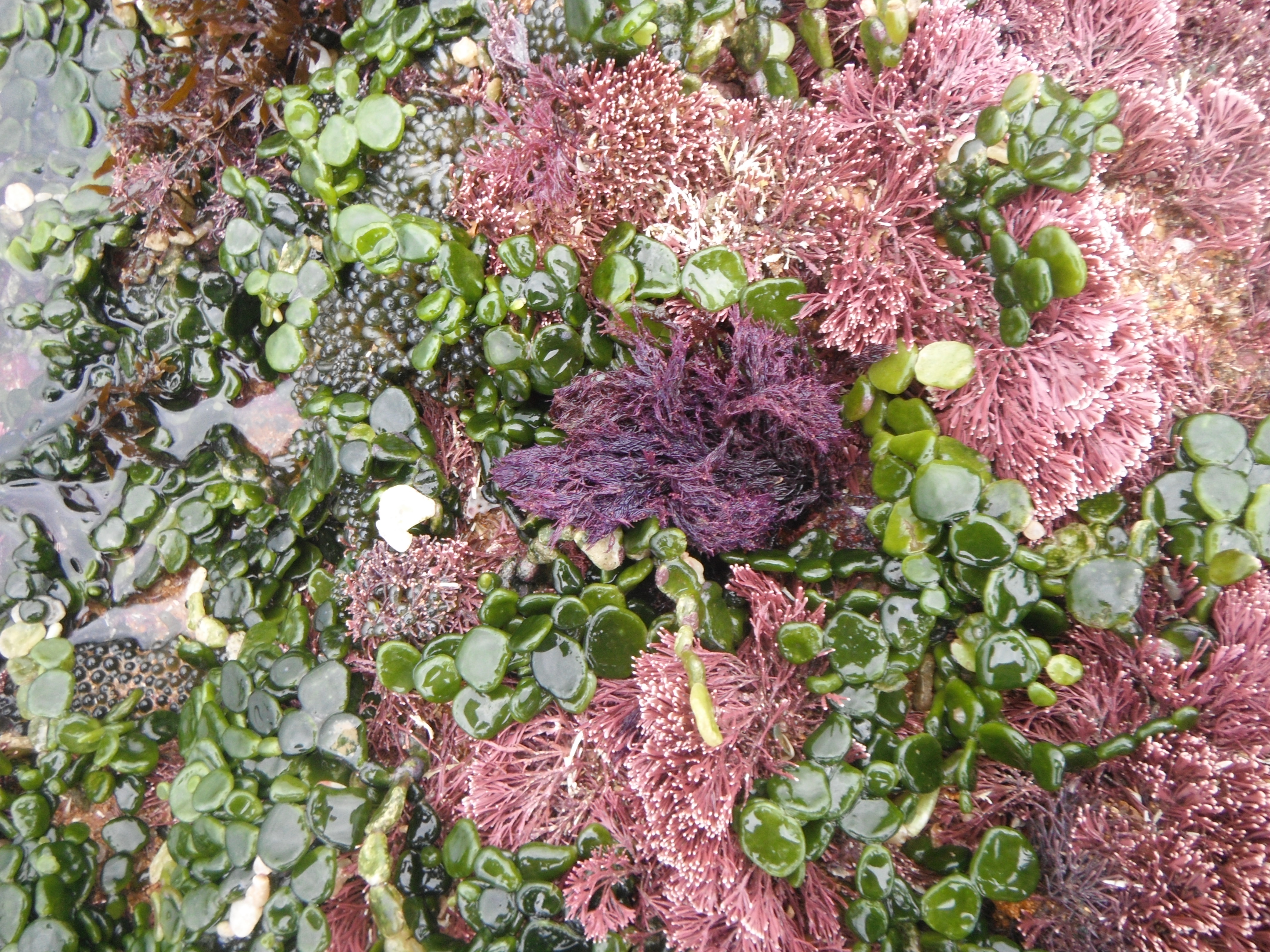

Supplement: Supplementary file 19 — “The development of research in diversity and biochemistry of seaweeds in intertidal areas is possible because of the wealth of shapes and colors and despite the simple thalloid organization of such bodies. It should be noted the ecological importance and fragility of macroalgae that however can develop into one of the most dynamic and complex ecosystems providing a beautiful natural spectacle.” Attribution: Levi Pompermayer Machado (Universidade Federal do Espírito Santo). [file 12898_2015_53_MOESM19_ESM.jpg]

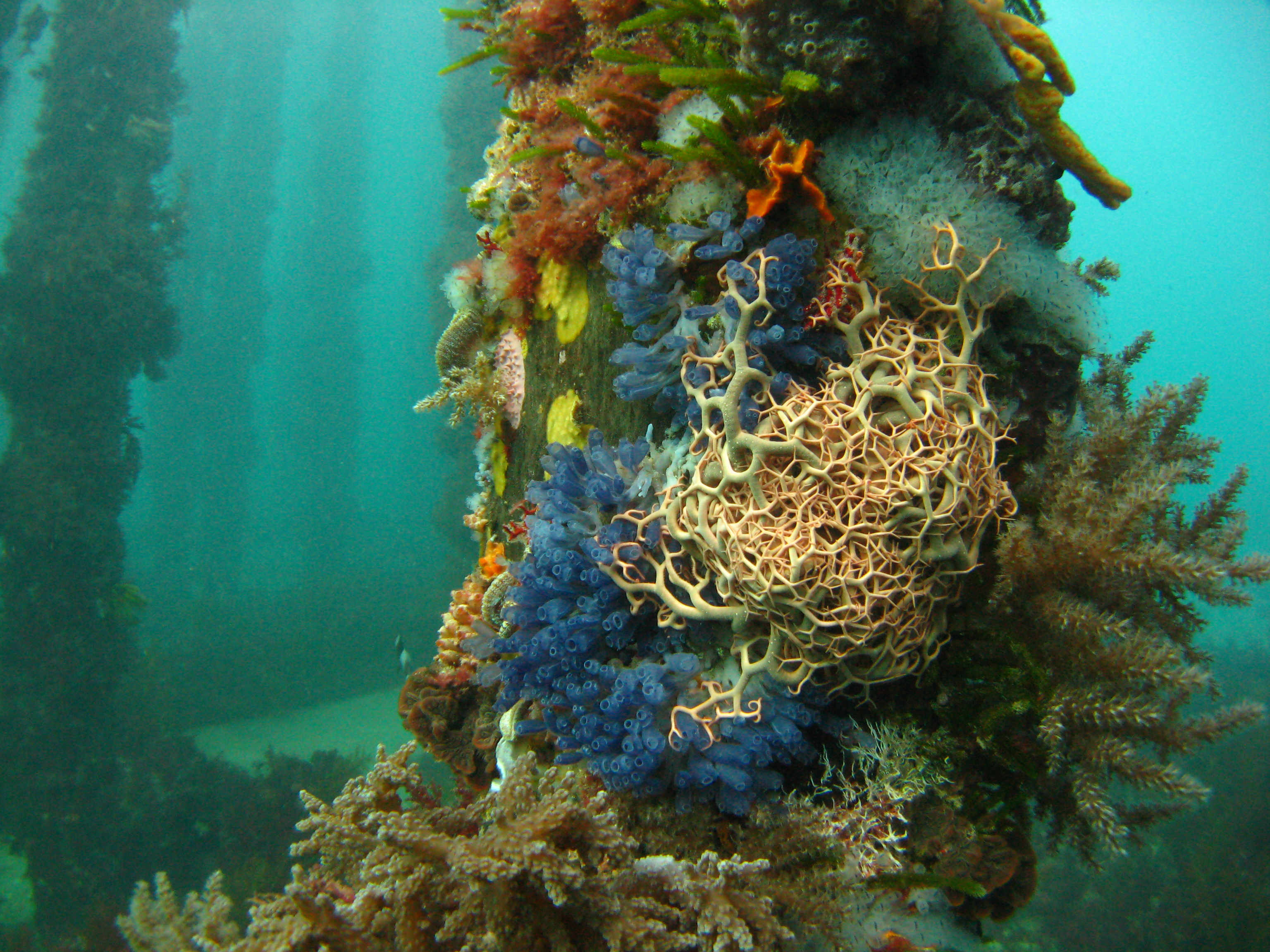

Supplement: Supplementary file 20 — Additional file 20: “South Australia’s secret gardens—this image was taken beneath the Stenhouse Bay jetty on the rugged coastline of Yorke Peninsula, Australia. A beautiful and fragile basket star (Astroboa ernae) shares a pylon with a colony of blue ascidians (Clavelina molluccensis), sponges, brown and red algae as well as a multitude of other species. Temperature marine habitats can be stunningly beautiful and support high levels of biodiversity. These cold water gardens like their coral reef equivalents are threatened by human development and climate change so an improved understanding their ecology is vital for conservation.” Attribution: Daniel Gorman (Universidade de São Paulo). [file 12898_2015_53_MOESM20_ESM.jpg]

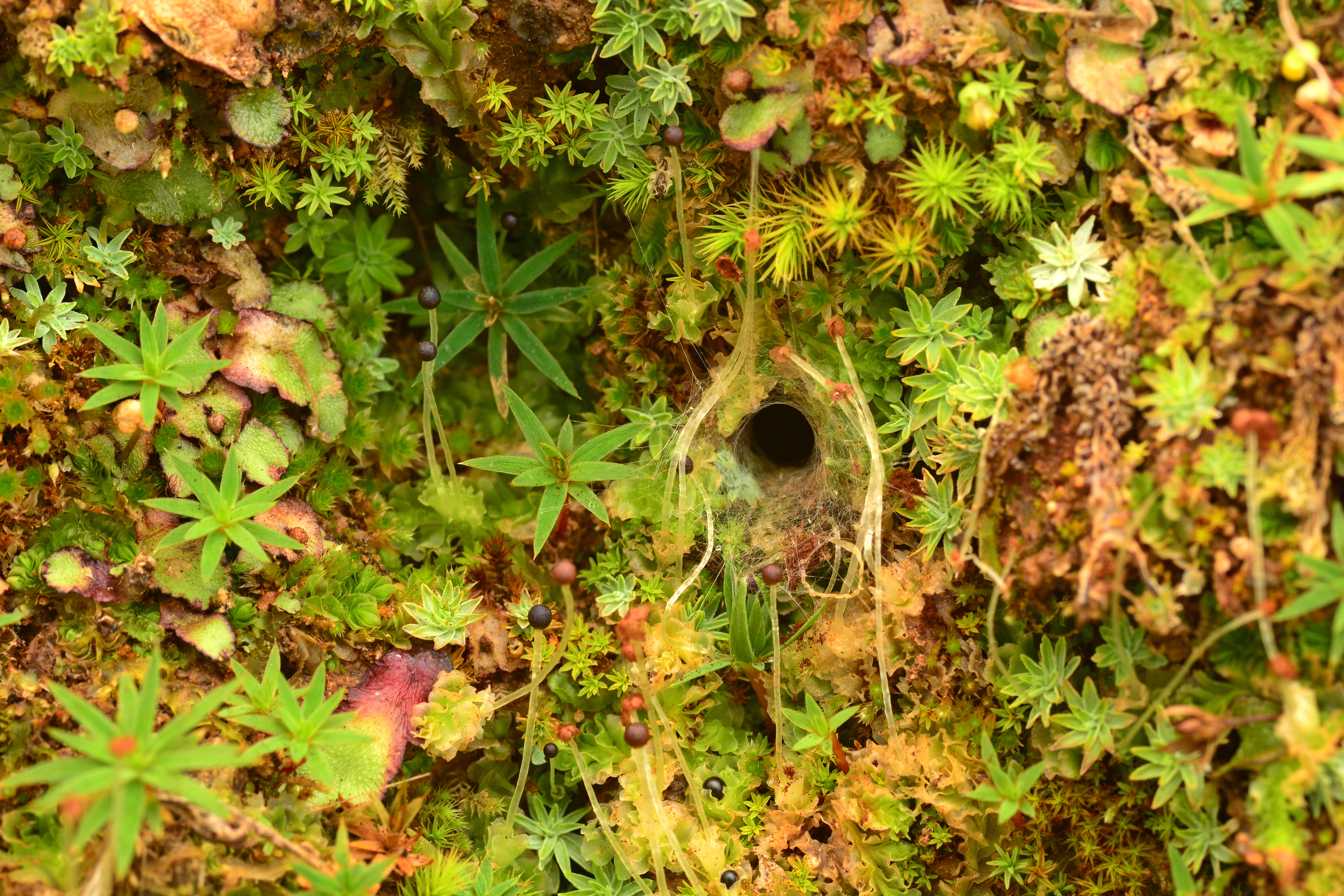

Supplement: Supplementary file 21 — “The spider’s garden. This image shows the web of a tube-dwelling spider (Ariadna sp.), in a marvelous, high diversity garden of bryophytes in Altos de Cantillana, Central Chile. This place is a high diversity and high endemism area of bryophytes.” Attribution: Bernardo Segura (Universidad de Chile). [file 12898_2015_53_MOESM21_ESM.jpg]

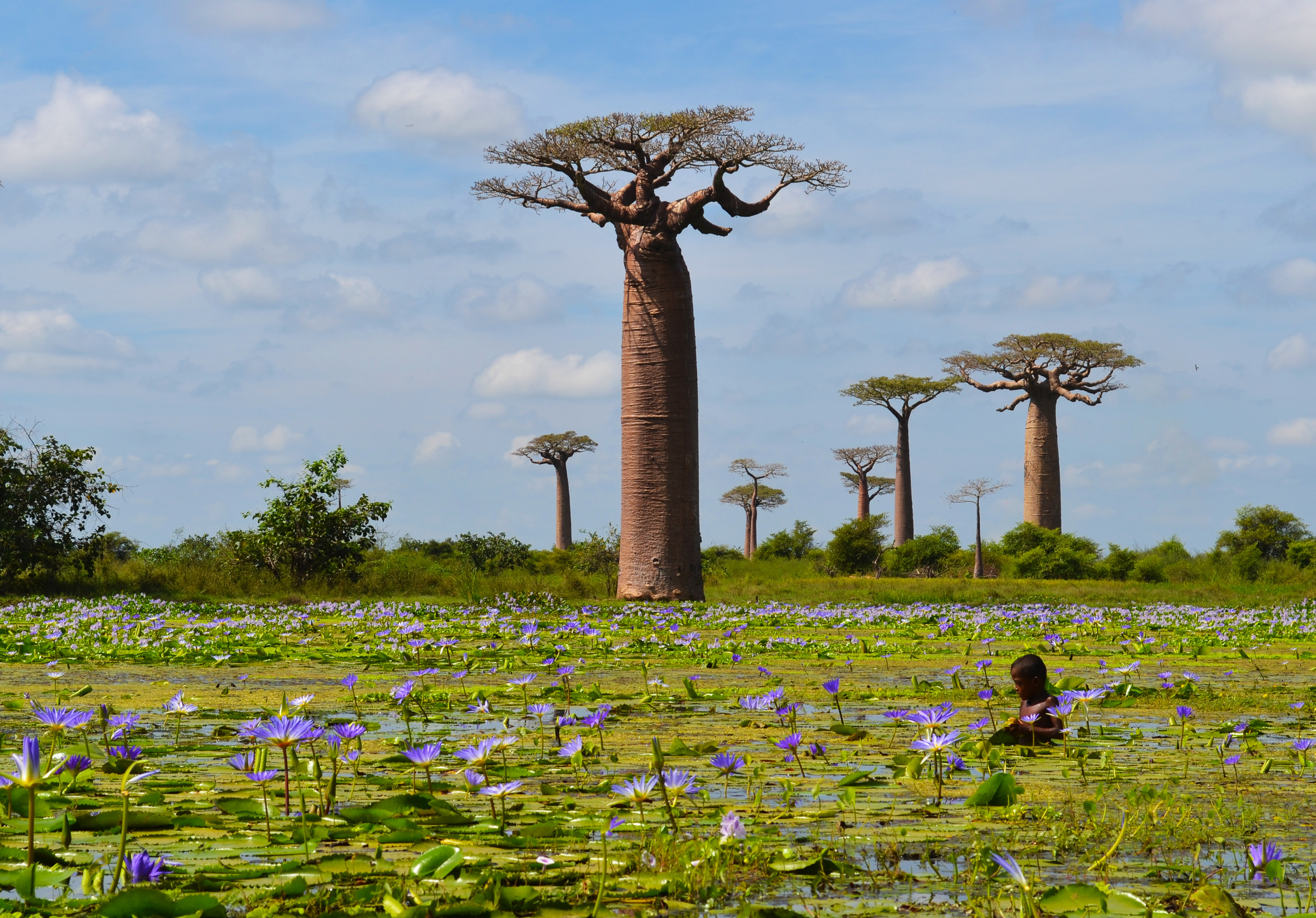

Supplement: Supplementary file 22 — “Along a dirt road in western Madagascar stands a grove of baobab trees (Adansonia grandidieri) collectively known as baobab alley. These trees are endemic to Madagascar and they form a striking landscape that draws travelers from around the world. The area is surrounded by stands of shallow water filled with lilies, where local children collect fallen baobab fruits. Local people call the baobab tree ‘renala’ meaning ‘mother of the forest.’” Attribution: Kathryn M. Everson (University of Alaska Museum). [file 12898_2015_53_MOESM22_ESM.jpg]

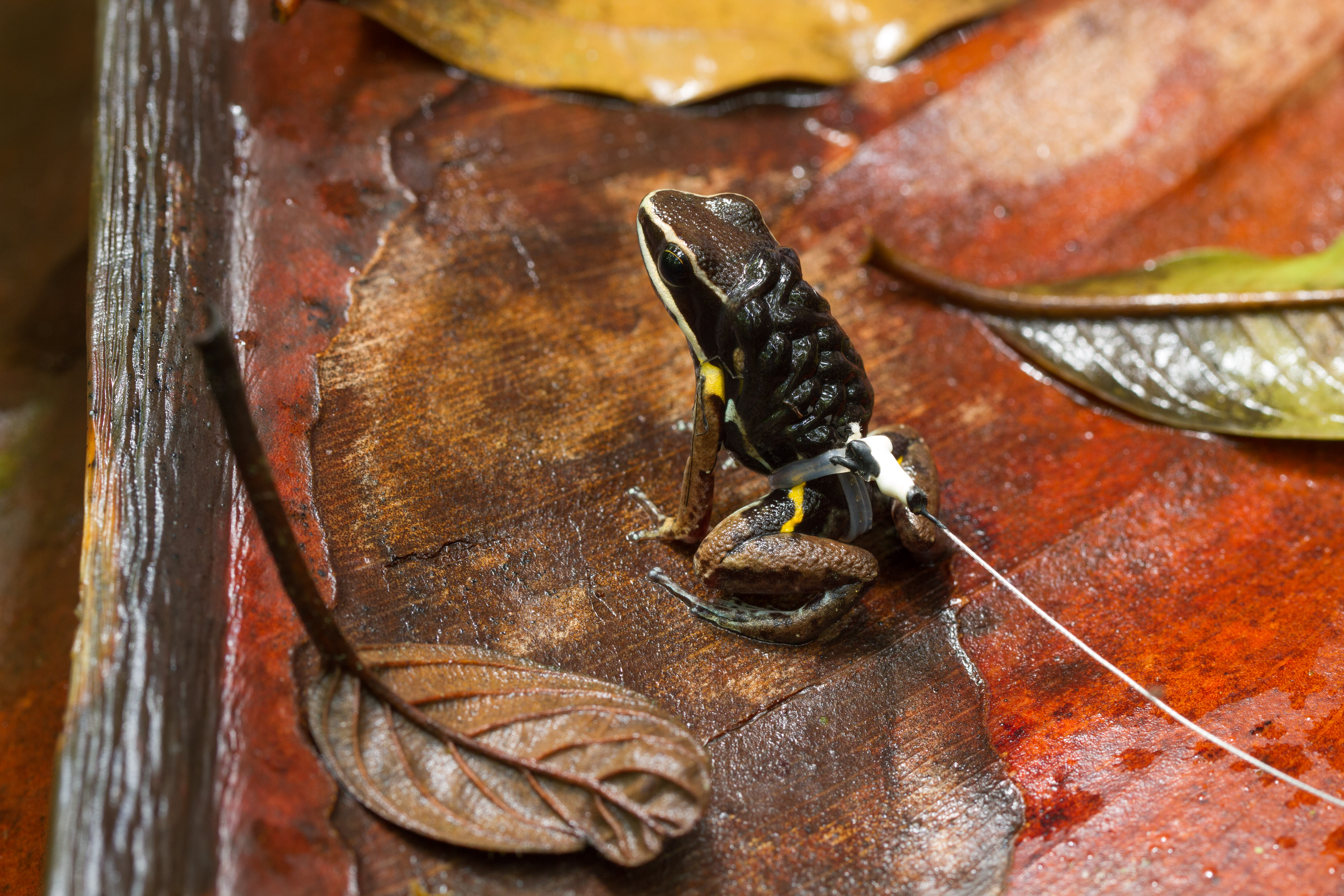

Supplement: Supplementary file 23 — “Father brilliant-thighed poison frog (Allobates femoralis) transporting his tadpoles while wearing a tracking device. Neotropical poison frogs (Dendrobatidae) show some of the most complex social and spatial behaviors among amphibians. In many species males shuttle tadpoles on their back from terrestrial territories to small ephemeral pools spread wide and far around the rain forest. We study the movement patterns (where they go) and the orientation mechanisms (how they find their way around) of poison frogs in the Amazon. We use miniature tracking devices to quantify the movement patterns of these small frogs in the field. Our findings are revealing a complex movement ecology and suggesting highly developed spatial learning ability in poison frogs. In this photograph, you see one of the male frogs being tracked at our field site in French Guiana while carrying his offspring to the water. The photograph actually captures the last moments of his life. Not long after I took this picture the frog was predated by a snake. Luckily, this happened right after he completed his parental duties and released his tadpoles in the water so his genes live on. While unfortunate for the frog, such events provide us unique insights into complex life histories of these small rain forest inhabitants.” Attribution: Andrius Pašukonis (University of Vienna). [file 12898_2015_53_MOESM23_ESM.jpg]
